# Combined inhibition of dopamine D1/D2 receptors induces cognitive and emotional dysfunction through oxidative stress and dopaminergic neuron damage

**DOI:** 10.3389/fnbeh.2025.1621017

**Published:** 2025-08-04

**Authors:** Xue Li, Yao Zhuang, Ya Ru Zhang, Ke Ke Fan, Xin Xin Chen, Xin Xing Chen, Xuan Yi Liu, Jing Sun, Li Liu

**Affiliations:** ^1^School of Medical and Health Engineering, Changzhou University, Changzhou, Jiangsu, China; ^2^School of Pharmacy & School of Biological and Food Engineering, Changzhou University, Changzhou, Jiangsu, China

**Keywords:** dopamine receptor inhibitors, monoamine oxidase B, reactive oxygen species, tyrosine hydroxylase, ethology

## Abstract

**Introduction:**

Dopamine system dysfunction is closely associated with nervous system diseases such as Parkinson’s disease and psychiatric disorder. Current research is limited to the individual application of dopamine D1 and D2 receptor-related agents, and the systematic effects of combined dopamine D1/D2 receptor inhibition on neural function remain unclear. In this study, we aimed to investigate the dose-dependent effects of co-DR1/2I (combined administration of dopamine receptor 1 inhibitor SCH39166 and dopamine receptor 2 inhibitor raclopride) on oxidative stress, learning, memory, emotion, and motor function in the substantia nigra, striatum, and hippocampus of mice.

**Methods:**

After administering varying doses of co-DR1/2I through gastric tubes to male C57BL/6 mice, we used enzyme-linked immunosorbent assay to measure monoamine oxidase B (MAO-B), reactive oxygen species (ROS), and superoxide dismutase (SOD) activity. Behavioral changes were assessed, using open field, rotarod, and water maze tests. Tyrosine hydroxylase positive neurons were labeled with immunofluorescence, and tyrosine hydroxylase levels were detected by Western blot (WB) assay.

**Results:**

Low-dose co-DR1/2I significantly increased MAO-B and ROS levels (*p* < 0.01) and decreased SOD activity (*p* < 0.01) in the substantia nigra, striatum, and hippocampus. MAO-B activity positively correlated with ROS (*r* = 0.916, *p* < 0.001) and negatively correlated with SOD (*r* = −0.685, *p* < 0.001), whereas ROS negatively correlated with SOD (*r* = −0.661, *p* < 0.001) in co-DR1/2I-treated mice. The medium- and high-dose groups exhibited spatial memory impairment (longer escape latency, *p* < 0.05) in the water maze and more anxiety-like behavior (reduced central zone time, *p* < 0.01) in the open field test; however, no abnormalities in motor coordination were observed in the rotarod test (*p* > 0.05). Immunofluorescence and WB confirmed a reduction in the dopaminergic neuron count after co-DR1/2I.

**Conclusion:**

This is the first study to demonstrate that co-DR1/2I triggers cognitive and emotional dysfunction by exacerbating oxidative stress and dopaminergic neuronal damage, thereby advancing our understanding of the neurotoxic mechanisms of dopamine receptor antagonists. Future studies are needed to explore targeted antioxidant therapies and receptor-selective modulation strategies to reduce the side effects.

## 1 Introduction

The dopamine system plays a crucial role in regulating motor control, cognitive function, and emotional behavior, and its dysfunction is closely related to various neurological diseases, such as Parkinson’s disease and schizophrenia (Grace, 2016; Latif et al., 2021). D1 and D2 receptors, the main mediators of dopaminergic signaling, are essential for maintaining nervous system function. Abnormalities in dopamine receptors not only affect the basal ganglia circuitry but may also participate in emotional and cognitive regulation through the limbic system (Gerfen and Surmeier, 2011; Howes et al., 2017, Prieto, 2017; Wang et al., 2021). However, the mechanisms and roles of dopamine receptors in neurodegenerative and psychiatric diseases are still not fully understood, limiting the development of drugs and treatment strategies for related diseases.

Current research mainly focused on the functional analysis of single dopamine receptor subtypes or isolated studies in specific brain regions. D2 receptor antagonists are widely used to treat psychiatric disorders, gastrointestinal diseases, and endocrine disorders (Kaar et al., 2020), whereas D1 receptor antagonists have shown limited clinical use. However, there are natural D1 receptor antagonists, including compounds found in plants such as soybeans and corn, which are susceptible to *Fusarium*, whose metabolites can inhibit the function of dopamine D1 receptors (Yuhong, 2001). Reports have also documented human infections with *Fusarium* affecting the function of the nervous system (Kościelecka et al., 2023; Kuæ-Szymanek et al., 2025), suggesting that the possibility of combined D1/D2 receptor antagonism in humans under certain circumstances cannot be ruled out. Although D1 and D2 receptor agonists have been confirmed to have neuroprotective or pro-oxidative stress effects (Ryzhova et al., 2020; Juza et al., 2023), systematic studies on the combined regulation of D1/D2 receptors are lacking, particularly regarding the synergistic regulation of the substantia nigra-striatum pathway and hippocampal function. Furthermore, previous experiments have often used acute dosing models, making it difficult to reflect the cumulative effects of long-term receptor inhibition on neural function, leading to a translational gap between preclinical research and actual drug treatment effects.

Currently, three key scientific questions remain unresolved in this field: first, does the combined inhibition of D1/D2 receptors produce synergistic or antagonistic neurobiological effects; second, are there differences in sensitivity to dopamine receptor inhibition across different brain regions; and third, can oxidative stress indicators serve as early biomarkers for predicting neural functional impairment. The answers to these questions will deepen our understanding of the regulatory mechanisms of the dopaminergic system and provide a theoretical basis for developing new neuroprotective strategies.

This study used molecular biology, behavioral, and histological methods to systematically assess the regulatory effects of combined dopamine D1/D2 receptor antagonists on the nervous system. The experimental design established a gradient dosing regimen to clarify the dose-response relationship, synchronously measuring oxidative stress markers in the substantia nigra, striatum, and hippocampus. A long-term dosing model was used to simulate clinical medication scenarios. Notably, this study evaluated dopaminergic neuronal function in the hippocampus, providing a new perspective for understanding the role of the dopamine system in cognitive dysfunction.

Using male C57BL/6 mice, we administered compound formulations of SCH39166 (a D1 antagonist) and raclopride (a D2 antagonist) via gastric gavage. The experimental plan included four key components: (1) quantifying monoamine oxidase B (MAO-B), reactive oxygen species (ROS), and superoxide dismutase (SOD) activity through enzyme-linked immunosorbent assay (ELISA) to assess oxidative stress levels; (2) using the Morris water maze and open field tests to detect learning, memory, and anxiety-like behaviors; (3) evaluating motor coordination through the rotarod test; and (4) Use immunofluorescence technology and WB experiment to detect the content of TH protein. This study elucidated the regulatory patterns of combined dopamine receptor inhibition in the oxidative stress network across multiple brain regions, the characteristic effects of different antagonist doses on cognitive and emotional behaviors, and the mediating role of dopaminergic neuron damage in these processes. These findings provide important experimental evidence for the optimization of dopamine-targeted therapeutic strategies.

## 2 Materials and methods

### 2.1 Mice

Mice were purchased from Nanjing Qingzilun Technology Co., Ltd. All experiments were conducted in accordance with the Guidelines for the Care and Use of Laboratory Animals published by the National Institutes of Health [Approval no. L20210226140 (March 2, 2021)] and the ethical regulations of Ethics Committee of Experimental Animal Center of Changzhou University. Owing to the neuroprotective effect of estrogen and its influence on the synthesis, release, and metabolism of dopamine, as well as its ability to regulate the expression and function of dopamine receptors (Shulman, 2002), we selected male mice. All male wild-type C57BL/6J mice (6 weeks old, weighing 22 ± 1.35 g) were housed in pathogen-free transparent cages with constant humidity (45%–65%) and temperature (18°C–26°C), with food (purchased from Xietong Biology) and water provided on a 12 h light/dark cycle.

### 2.2 Experimental grouping and preparation of mice exposed to co-DR1/2I

**FIGURE 1:**
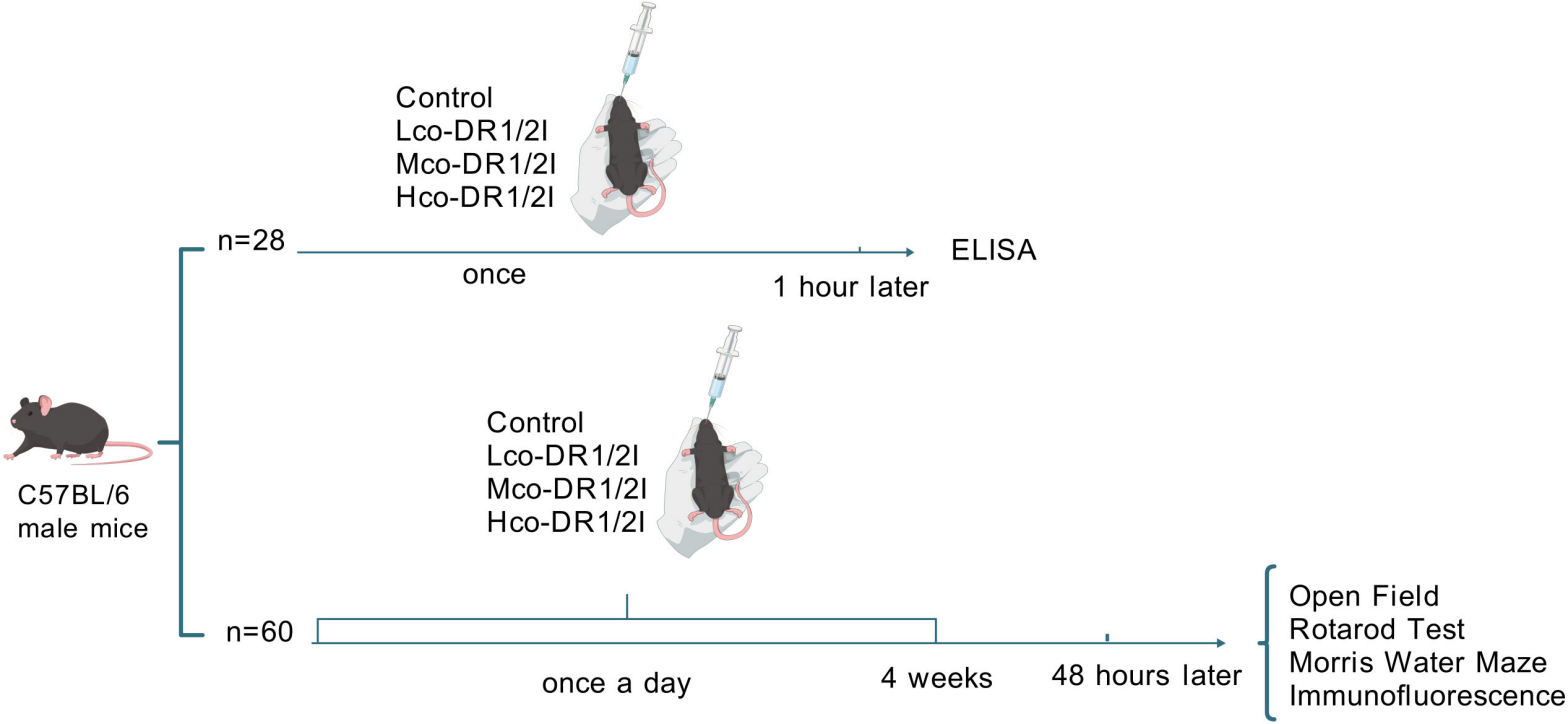
Experimental procedure. There were two parts in the experiment. Part 1: the control group (*n* = 7) received equivalent normal saline; Lco-DR1/2I (*n* = 7) received low-dose of co-DR1/2I; Mco-DR1/2I (*n* = 7) received medium-dose of co-DR1/2I; Hco-DR1/2I (*n* = 7) received high-dose of co-DR1/2I. Part 2: the control group (*n* = 15) received equivalent normal saline once a day; Lco-DR1/2I (*n* = 15) received low-dose of co-DR1/2I once a day; Mco-DR1/2I (*n* = 15) received medium-dose of co-DR1/2I once a day; Hco-DR1/2I (*n* = 15) received high-dose of co-DR1/2I once a day. The medication would last for 4 weeks; behavioral training and testing would begin 48 h after discontinuation. Finally, immunofluorescence was performed.

Overall, 28 male C57BL/6 mice (6 weeks old) were randomly categorized into four groups according to the random number table method: control, co-DR1/2I low, medium, and high dose groups. The control group was administered saline, while the other three groups were administered low, medium, or high doses via gavage (SCH39166: 0.025 mg/kg, 0.05 mg/kg, 0.1 mg/kg; raclopride: 0.25 mg/kg, 0.5 mg/kg, 1 mg/kg). Since the drug’s effect reaches its peak within 1–2 h, the mice were euthanized by cervical dislocation 1 h after administration, and their brains were collected to measure the levels of MAO-B, ROS, and SOD activity in the substantia nigra, striatum, and hippocampus using ELISA. An additional 60 male C57BL/6 mice (6 weeks old) were randomly categorized into four groups: control, co-DR1/2I low, medium, and high dose group. The control group was administered saline, while the other three groups were administered low, medium, or high doses via gavage (SCH39166: 0.025 mg/kg, 0.05 mg/kg, 0.1 mg/kg; raclopride: 0.25 mg/kg, 0.5 mg/kg, 1 mg/kg) once a day for 4 weeks. All four groups were maintained under the same feeding conditions, and behavioral tests were conducted after 4 weeks. After testing, the mice were perfused, and brain tissue slices were analyzed for TH content using immunofluorescence. SCH39166 and risperidone were purchased from MedChemExpress. The procedure is shown in Figure 1.

### 2.3 ELISA experiment

After cervical dislocation, the brains were quickly removed, and the substantia nigra, striatum, and hippocampus were isolated. The brain tissue was thoroughly homogenized on ice, and the homogenate was centrifuged at 5000 *g* for 10 min at 4°C. The supernatant was collected, and the levels of MAO-B, ROS, and SOD activity in the supernatant were measured using ELISA kits (mouse monoamine oxidase activity assay kit TW63204H, mouse reactive oxygen assay kit TW6398H, and mouse superoxide dismutase assay kit TW12776), all purchased from Shanghai Tongwei Company.

### 2.4 Open field experiment

An open-field apparatus was purchased from RWD Life Sciences. The square uncovered plastic box measured 40 cm × 40 cm × 40 cm, with white walls and a bottom. The bottom was virtually categorized into equal-area grids and illuminated uniformly at 60 lx. A camera was installed at the top and connected to video tracking software SmartV3.0, which automatically recorded the movement trajectory. The experiment was conducted in a soundproof, interference-free environment with a background noise below 40 dB. The temperature was maintained at 22°C ± 2°C and humidity at 50% ± 10%. The mice were gently placed in the center of the open field box, and the video recording system was activated. The mice were allowed to explore freely for 10 min while the experimenter left the room to avoid interference.

### 2.5 Fatigue rotarod experiment

A ZB-200 fatigue rotarod apparatus was purchased from TaiMeng company. Adaptation training: Each mouse underwent one session of adaptation training daily for 3 days before the formal experiment. The animals were placed on a stationary rotarod to explore freely for 5 min, followed by rotation at a constant speed (5 rpm) for 2 min to establish motor memory.

Formal experiment: The initial rotation speed was set to 4 rpm and linearly accelerated to 40 rpm at a rate of 0.5 rpm/s. The timing was stopped when the animal completely fell into the bottom-sensing area, and the latency was recorded. Each group of animals underwent three repeated tests, with a 30 min interval between each test, and the average value was taken as the final result.

### 2.6 Water maze experiment

The water maze apparatus was purchased from RWD Life Sciences. The experiment comprised three phases: adaptation, training, and probing. In the adaptation phase (Day 1), the platform was removed, and each mouse swam freely for 60 s to familiarize itself with the environment; in the training phase (Days 2, 3, and 4), the pool was categorized into four quadrants, and the platform was fixed in a target quadrant. Each training session involved placing the mouse in different quadrants. If the mouse found the platform and stayed there for more than 10 s within 60 s, the training was stopped. If the time limit was exceeded, the mouse was gently guided to the platform and allowed to remain there for 15 s. A minimum interval of 30 min was observed between training sessions; in the probe phase (Day 5), the platform was removed, and the mouse was placed in the water from a quadrant far from the original platform, swimming freely for 60 s. The time spent in the original platform quadrant and the number of crossings over the original platform location were recorded. After each swim, the mice were immediately dried with a towel and placed on a 37°C heating pad to prevent cold stress.

### 2.7 Tissue section immunofluorescence experiment

After the behavioral experiments, six mice from each group were intraperitoneally injected with pentobarbital sodium (45 mg/kg) for anesthesia. After anesthesia, the mice were fixed in position, and the chest cavity was opened to expose the heart. A perfusion needle was inserted into the left ventricle, and physiological saline was perfused from the left ventricle using a BT-100 peristaltic pump. The right atrium was cut to drain blood until the liver appeared grayish-white, and 4% formaldehyde was used for perfusion until the limbs and tail became stiff and straight, respectively. Brain tissue was gently removed, fixed in 4% paraformaldehyde for 24 h, and dehydrated using a gradient of 20% and 30% sucrose. Coronal sections were made using a cryostat; after washing with PBS, the sections were placed in sodium citrate buffer; 10% donkey serum (Servicebio) was used for blocking at room temperature for 30 min; diluted primary antibody (Servicebio) was added and incubated at 4°C for 24 h; after three washes with PBS, the secondary antibody (Servicebio) was added and incubated at room temperature in the dark for 50 min. DAPI (Servicebio) was used for nuclear staining for 10 min, anti-fluorescence quenching mounting medium was used, and images were observed and collected under a fluorescence microscope (Nikon Eclipse C1).

### 2.8 Western blot

Radioimmunoassay assay buffer (RIPA) lysis buffer (Biyuntian) was added to the substantia nigra, striatum, and hippocampus tissues to extract total protein. The protein concentration was detected by BCA method. 30–50 μg of protein was loaded, and 5% concentrated gel and 12% separation gel were prepared. After protein separation, the protein was transferred to a PVDF membrane, sealed with 5% milk powder for 2 h, and incubated with 1:2000 antibody (Proteintech) overnight at 4°C. The next day, 1:1000 secondary antibody (Biyuntian) was added and incubated at room temperature for 1 h, followed by washing with TBST. The imaging is performed by gel imaging analyzer and analyzed by ImageJ software.

### 2.9 Statistical methods

Data were analyzed using SPSS 26.0 software, and results are expressed as mean ± standard deviation. Comparisons between groups were performed using a one-way analysis of variance. The SmartV3.0 video analysis system was used to analyze the results of the water maze and open-field experiments. Statistical significance was set at *p* < 0.05. Graphing software included Graphpad Prism 10.1.2 and Photoshop 2024.

## 3 Results

### 3.1 Effects of co-DR1/2I on MAO-B, ROS, and SOD activity in the substantia nigra, striatum, and hippocampus of mice

Regarding the ELISA experiment, one-way ANOVA showed that after co-DR1/2I administration, the MAO-B activity in the tested brain regions of mice increased, with the low-dose group showing the most significant increase, followed by the medium dose group: substantia nigra (*F* = 558.84, *p* < 0.01), striatum (*F* = 115.06, *p* < 0.01), and hippocampus (*F* = 142.02, *p* < 0.01). ROS activity also increased in all groups, with the low-dose group showing the most significant increase, followed by the medium dose group: substantia nigra (*F* = 361.24, *p* < 0.01), striatum (*F* = 91.37, *p* < 0.01), and hippocampus (*F* = 316.38, *p* < 0.01). In addition, SOD activity decreased, with the low-dose group showing the most significant decrease, followed by the medium dose group: substantia nigra (*F* = 18.67, *p* < 0.01), striatum (*F* = 17.76, *p* < 0.01), and hippocampus (*F* = 232.08, *p* < 0.01), as presented in Table 1.

**TABLE 1:** Monoamine oxidase B (MAO-B), ROS, and SOD activities in substantia nigra, corpus striatum and hippocampus.

Sample	Group	MAO-B	*F*	*P*	ROS	*F*	*P*	SOD	*F*	*P*
SN	Control	123.55 ± 3.80	558.84	<0.01	49.57 ± 5.32	361.24	<0.01	65.31 ± 6.17	18.67	<0.01
	Lco-DR1/2I	275.57 ± 7.46			145.09 ± 6.43			45.45 ± 4.56		
	Mco-DR1/2I	229.13 ± 10.91			122.44 ± 5.83			50.07 ± 5.11		
	Hco-DR1/2I	160.97 ± 4.79			75.3 ± 5.74			56.62 ± 4.36		
CPU	Control	132.78 ± 14.07	115.06	<0.01	51.76 ± 8.51	91.37	<0.01	64.82 ± 6.73	17.76	<0.01
	Lco-DR1/2I	286.73 ± 17.04			145.95 ± 12.20			45.08 ± 4.82		
	Mco-DR1/2I	235.47 ± 16.42			119.23 ± 10.89			48.78 ± 4.19		
	Hco-DR1/2I	161.33 ± 19.00			75.85 ± 12.93			54.57 ± 4.85		
Hi	Control	151.11 ± 4.55	142.02	<0.01	60.93 ± 6.95	316.38	<0.01	65.65 ± 7.37	232.08	<0.01
	Lco-DR1/2I	302.44 ± 21.43			153.96 ± 6.56			46.50 ± 4.73		
	Mco-DR1/2I	258.33 ± 14.58			125.51 ± 7.08			49.63 ± 4.55		
	Hco-DR1/2I	182.04 ± 13.00			84.43 ± 7.37			56.71 ± 4.49		

Lco-DR1/2I, low dose of co-DR1/2I; Mco-DR1/2I, medium dose of co-DR1/2I; Hco-DR1/2I, high dose of co-DR1/2I. SN, substantia nigra; CPU, corpus striatum; Hi, hippocampus. MAO-B, monoamine oxidase B; ROS, reactive oxygen species; SOD, superoxide dismutase.

**FIGURE 2:**
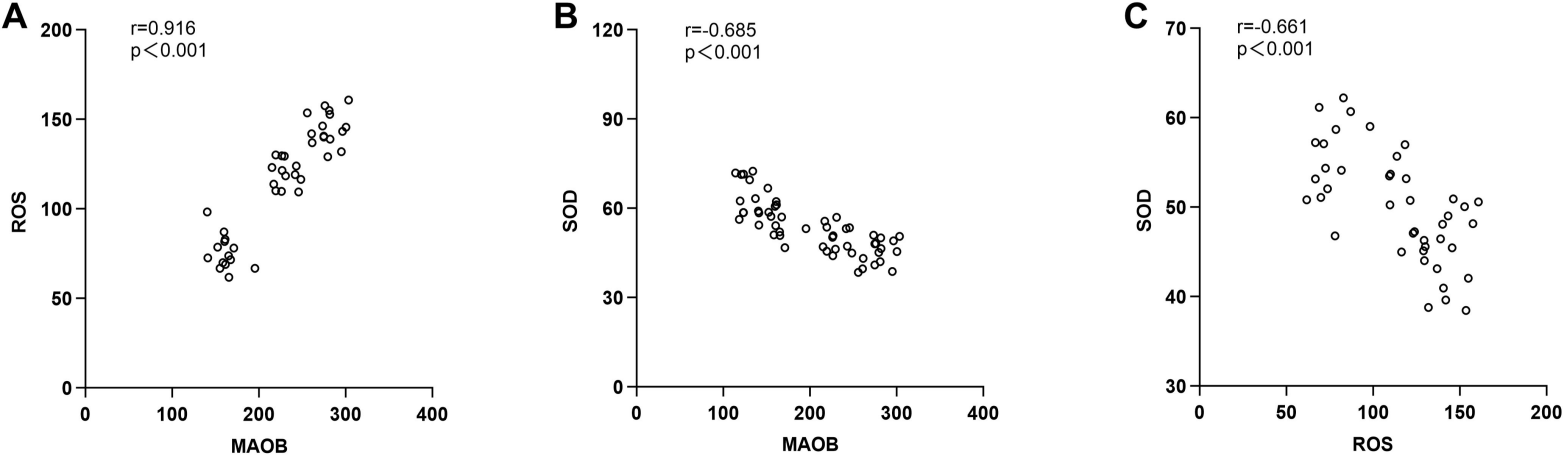
Correlation analysis of MAO-B, ROS and SOD. **(A)** Correlation between monoamine oxidase B activity and reactive oxygen species activity. **(B)** Correlation between monoamine oxidase B activity and superoxide dismutase activity. **(C)** Correlation between reactive oxygen species activity and superoxide dismutase activity.

In mice treated with co-DR1/2I, MAO-B activity was positively correlated with ROS (*r* = 0.916, *p* < 0.001), MAO-B activity was negatively correlated with SOD (*r* = −0.685, *p* < 0.001), and ROS activity was negatively correlated with SOD (*r* = −0.661, *p* < 0.001), as shown in Figure 2.

### 3.2 Effect of co-DR1/2I on the anxiety level of mice

**FIGURE 3:**
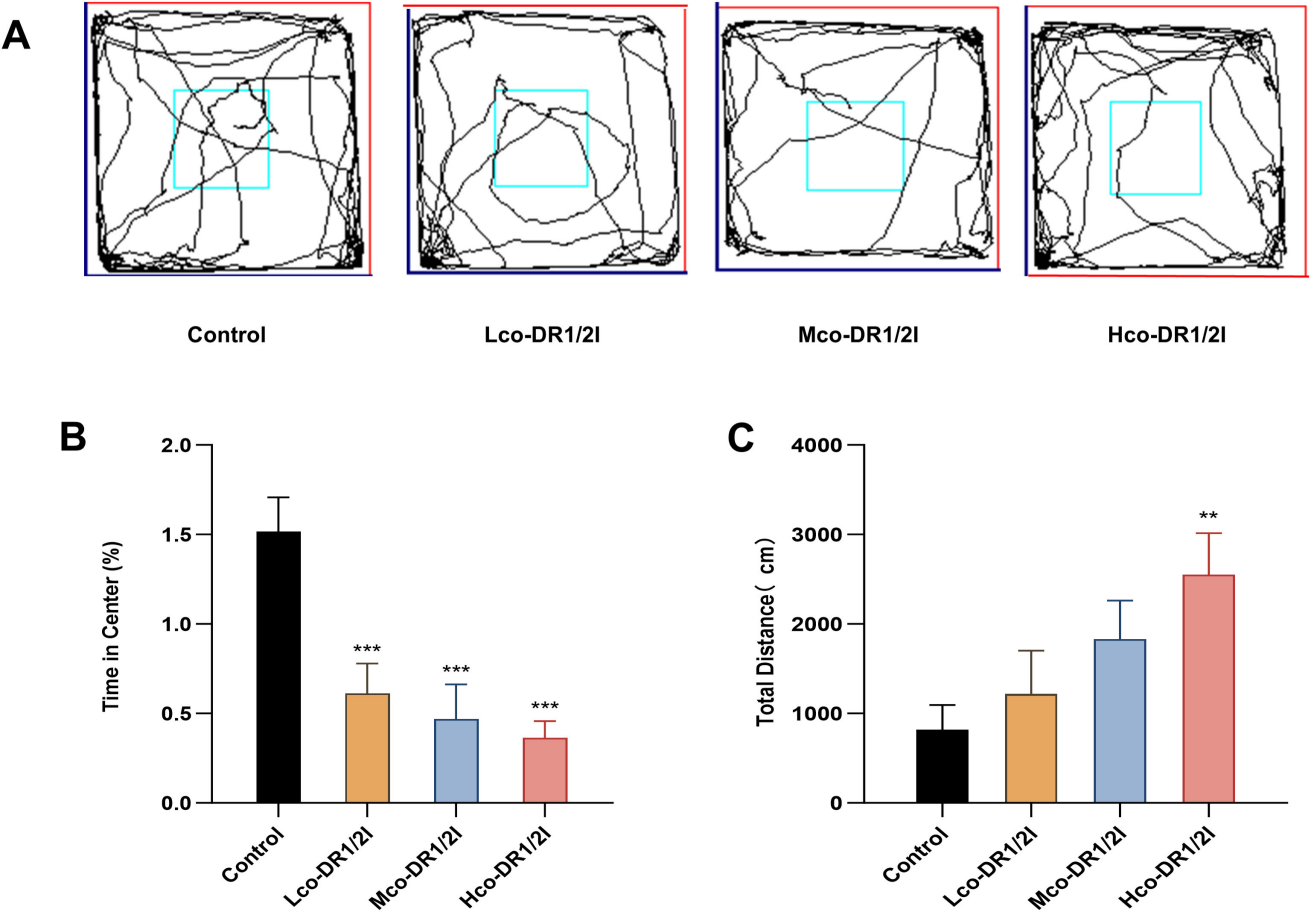
The effect of co-DR1/2I on the emotions of mice. **(A)** Locomotor activity trace of the mice in open field experiment. **(B)** Percentage of time spent in the central area of the open field compared to the total time spent. **(C)** Total distance traveled by the mice throughout the entire experimental period. **p < 0.01, ***p < 0.001, VS control group. Lco-DR1/2I, low dose of co-DR1/2I; Mco-DR1/2I, medium dose of co-DR1/2I; Hco-DR1/2I, high dose of co-DR1/2I.

In terms of the open field experiment, mice trajectory in the mice is shown in Figure 3A. The central zone stay time of mice treated with co-DR1/2I was reduced; the difference between the four groups was significant (*F* = 10.04, *p* < 0.001). Using LSD multiple comparisons test, significant differences between the Control and Lco-DR1/2I groups (*p* < 0.001) and Mco-DR1/2I group (*p* < 0.001) and Hco-DR1/2I group (*p* < 0.001) were found, as shown in Figure 3B. Mice treated with co-DR1/2I showed an increased total distance moved throughout the experimental area. Using ANOVA, the results revealed a significant difference between the four groups (*F* = 3.43, *p* < 0.05). Using LSD multiple comparisons test, a significant difference between the Control and Hco-DR1/2I groups (*p* < 0.01) was found, as shown in Figure 3C.

### 3.3 Effect of co-DR1/2I on the motor function of mice

**FIGURE 4:**
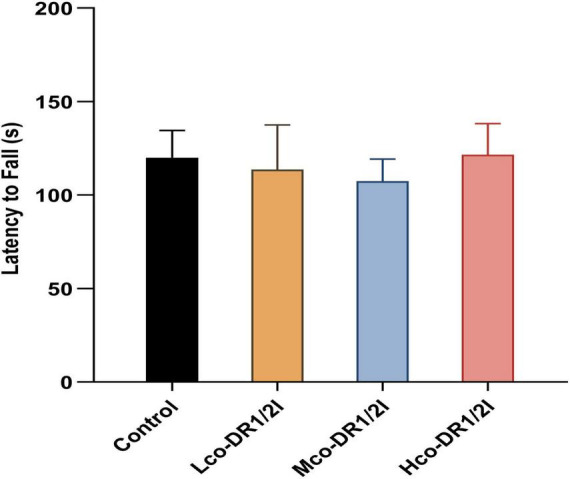
The effect of co-DR1/2I on motor function in mice. The time it takes for a mouse to start spinning and drop from the spinning stick. There was no statistically significant difference among the four groups. Lco-DR1/2I, low dose of co-DR1/2I; Mco-DR1/2I, medium dose of co-DR1/2I; Hco-DR1/2I, high dose of co-DR1/2I.

In the spinning rod experiment, there was no significant difference in motor function between mice using co-DR1/2I (*P* > 0.05), as shown in Figure 4.

### 3.4 Effects of co-DR1/2I on learning and memory abilities in mice

**FIGURE 5:**
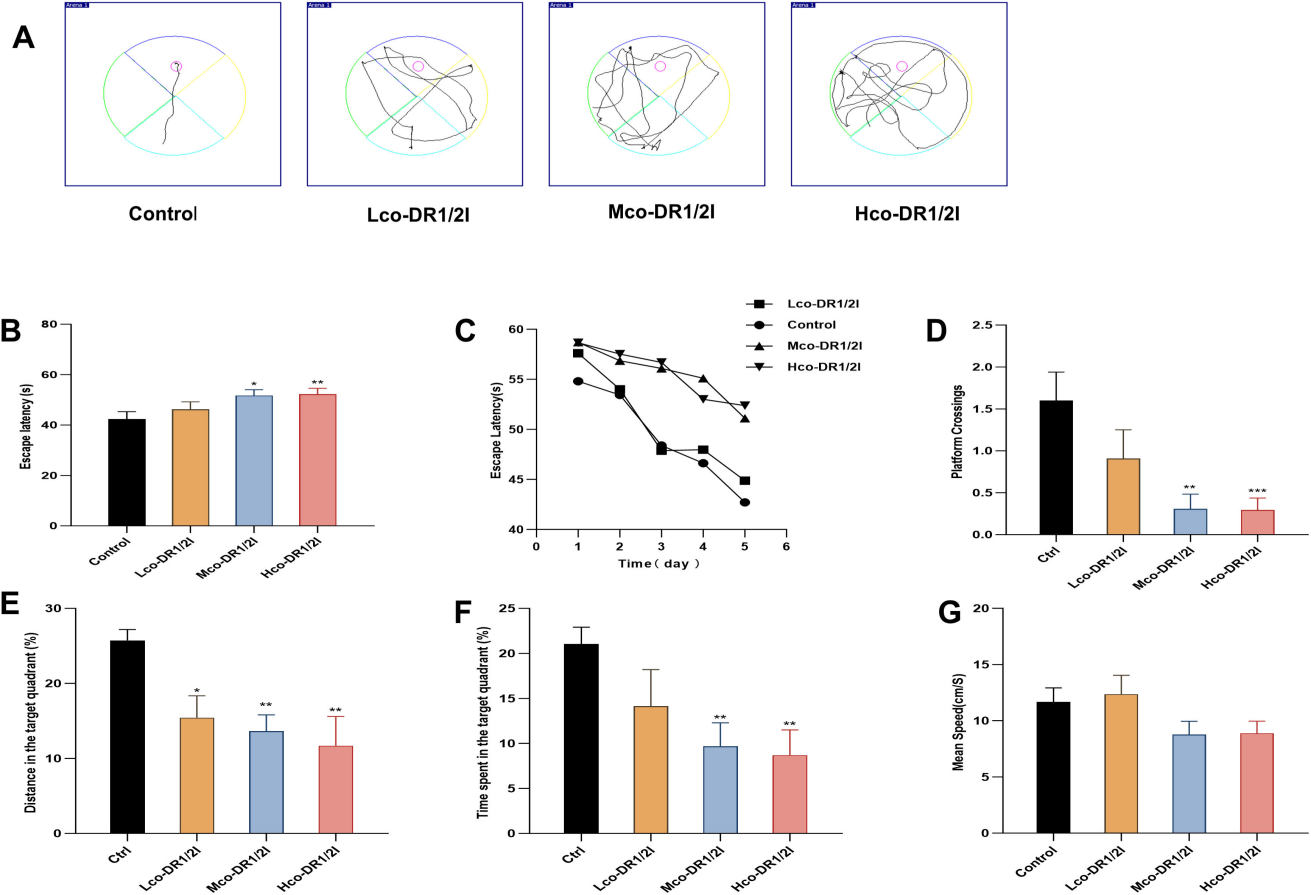
The effect of co-DR1/2I on learning and memory abilities in mice. **(A)** Locomotor activity trace of the mice in Morris water maze. **(B)** Time required for mice to enter the water and find the hidden platform. **(C)** Changes in latency during the 5-days training period. **(D)** Number of times the platform was crossed. **(E)** Percentage of movement distance within the target quadrant to the total movement distance of the entire experimental area. **(F)** Percentage of movement time within the target quadrant to the total movement time of the entire experimental area. **(G)** The mean speed of mice in the Morris water maze. *p < 0.05, **p < 0.01, ***p < 0.001, VS control group. Lco-DR1/2I, low dose of co-DR1/2I; Mco-DR1/2I, medium dose of co-DR1/2I; Hco-DR1/2I, high dose of co-DR1/2I.

Considering the Morris water maze, the trace of locomotor activity in the mice is shown in Figure 5A. The navigation experiment showed that the escape latency of the drug-treated mice was prolonged; using one-way ANOVA, a significant difference was found between the four groups (*F* = 3.31, *p* < 0.01). Using Fisher’s Least Significant Difference (LSD) multiple comparisons test, a significant difference between the Control and Mco-DR1/2I groups (*p* < 0.05) and Hco-DR1/2I group (*p* < 0.01) was found, as shown in Figure 5B. The training results from the first 5 days indicated that the escape latency of the mice gradually shortened over time, with the mice treated with dopamine inhibitors showing a smaller reduction in escape latency, as shown in Figure 5C. This suggests that co-DR1/2I affects spatial learning ability in mice.

In terms of the spatial exploration test, the number of crossings in the target quadrant was reduced in drug-treated mice; using ANOVA, a significant difference between the four groups was revealed (*F* = 5.94, *p* < 0.01). Using LSD multiple comparisons test, significant differences between the Control and Mco-DR1/2I groups (*p* < 0.01), and Hco-DR1/2I group (*p* < 0.001) were found, as shown in Figure 5D. The percentage of the total distance traveled in the target quadrant was lower in the drug-treated mice; the difference between the four groups was significant (*F* = 4.08, *p* < 0.05). Multiple comparisons indicated a significant difference between the Control and Lco-DR1/2I groups (*p* < 0.05) and Mco-DR1/2I group (*p* < 0.01) and Hco-DR1/2I group (*p* < 0.01), as shown in Figure 5E. The time spent in the target quadrant was reduced in drug-treated mice; the difference between the four groups was significant (*F* = 3.52, *p* < 0.05). Multiple comparisons revealed significant differences between the Control and Mco-DR1/2I group (*p* < 0.01) and Hco-DR1/2I group (*p* < 0.01), as shown in Figure 5F. There was no statistically significant difference in exercise speed between the four groups of mice (*F* = 2.289, *P* > 0.05), as shown in Figure 5G.

### 3.5 The effect of co-DR1/2I on dopamine neurons in mice

**FIGURE 6:**
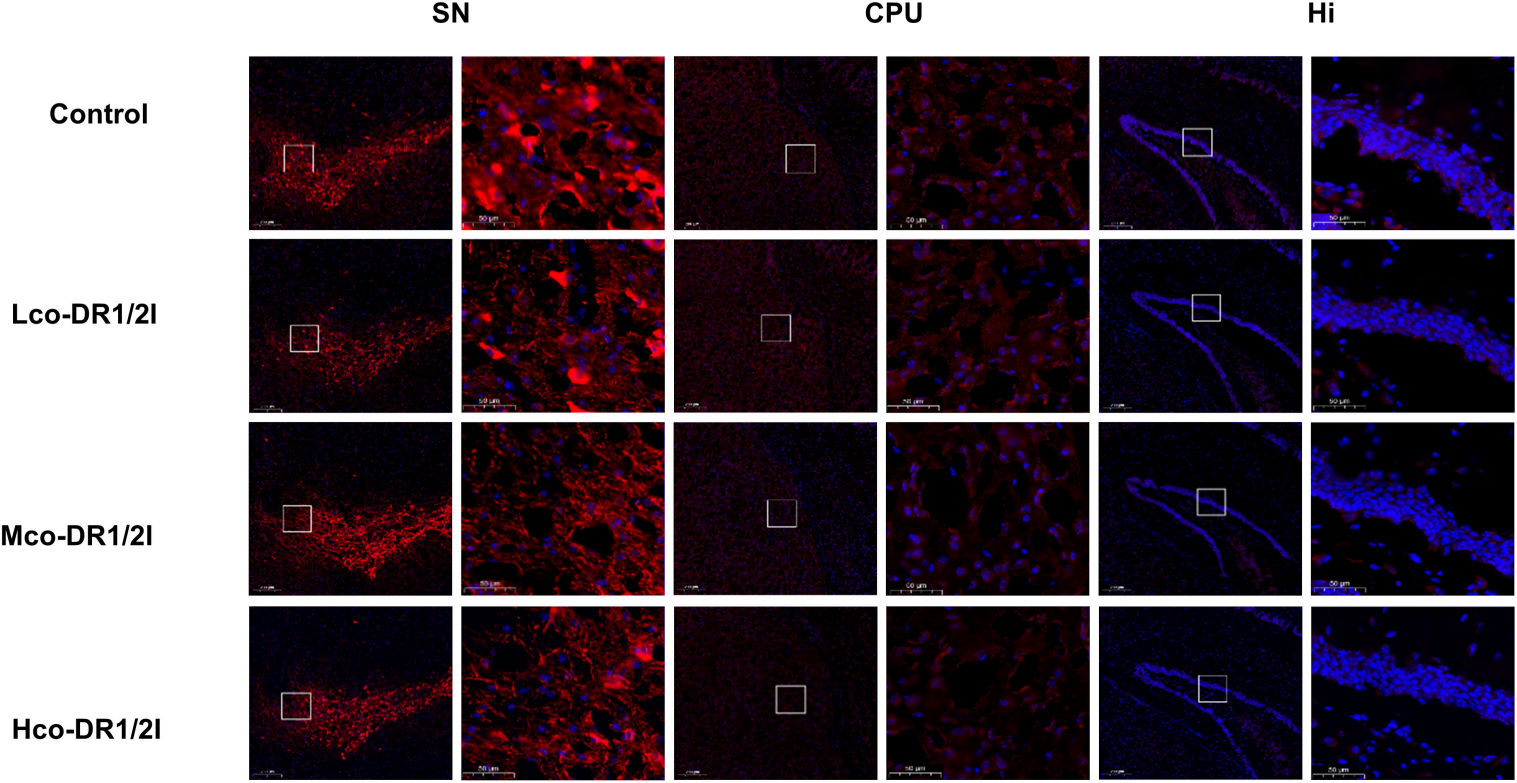
Results of immunofluorescence experiment. Immunofluorescence results of TH (red) in the SN, CPU, and Hi (scale bar = 200/50 um). SN, substantia nigra; CPU, corpus striatum; Hi, hippocampus. Lco-DR1/2I, low dose of co-DR1/2I; Mco-DR1/2I, medium dose of co-DR1/2I; Hco-DR1/2I, high dose of co-DR1/2I.

**FIGURE 7:**
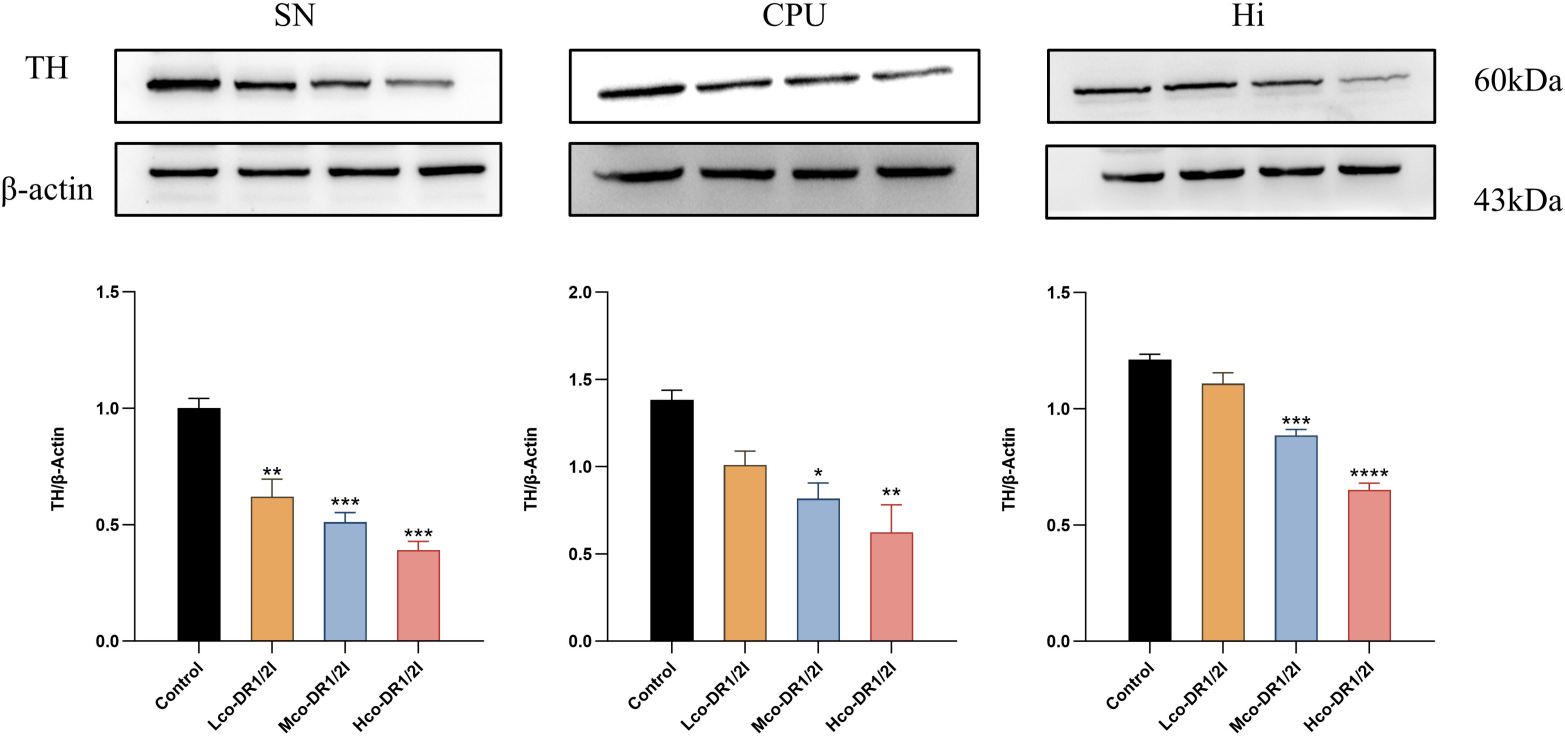
Content of tyrosine hydroxylase. SN, substantia nigra; CPU, corpus striatum; Hi, hippocampus. TH, tyrosine hydroxylase VS control group. Lco-DR1/2I, low dose of co-DR1/2I; Mco-DR1/2I, medium dose of co-DR1/2I; Hco-DR1/2I, high dose of co-DR1/2I. *p < 0.05, **p < 0.01, ***p < 0.001, ****p < 0.0001.

In the tissue of immunofluorescence slices, red light represents TH content, indirectly reflecting the number of dopaminergic neurons. The results showed that after using co-DR1/2I, TH positive neurons in the substantia nigra, striatum, and hippocampus of mice decreased, as shown in Figure 6. The WB results showed that after using co-DR1/2I, the TH levels in the substantia nigra, striatum, and hippocampus of mice were significantly reduced, as shown in Figure 7.

## 4 Discussion

Dopamine D1 receptor inhibitors are linked to cognitive function; however, they are not widely used clinically because of their adverse effects. Compared to inhibitors, D1 agonists are often used to improve PD motor symptoms; however, late-stage patients may develop D1 receptor hypersensitivity, and short-term antagonism may alleviate symptoms (Isaacson et al., 2023). Parkinson’s disease is accompanied by cognitive dysfunction and emotional disorders, which are closely related to abnormalities in the dopaminergic neural circuits of the hippocampus and limbic system (Rana et al., 2015). Currently, although drugs targeting dopamine receptor regulation can improve motor symptoms, their efficacy for cognitive and emotional disorders is limited, and long-term use may exacerbate neurodegenerative changes (Jing et al., 2023; Woitalla et al., 2023). Dopamine D2 receptor inhibitors also exhibit some neurological symptoms during use (Niemegeers, 1982), suggesting that the balance of dopamine D1/D2 receptors plays a complex role in the regulation of neural function.

In this study, we systematically evaluated the cross-brain area effects of dopaminergic system inhibition on the substantia nigra-striatum pathway and hippocampal function using a combination of D1/D2 receptor-specific inhibitors (SCH39166 and raclopride). The experiment employed a multidose gradient design combined with molecular biology detection (MAO-B/ROS/SOD), behavioral assessments (water maze, open field experiment), and immunofluorescence technology, revealing for the first time that the combined inhibition of D1/D2 receptors causes the loss of dopaminergic neurons and induces spatial memory impairment and anxiety-like behavior in a dose-dependent manner by exacerbating oxidative stress damage.

Research has shown that neurons in the hippocampus exhibit significant sensitivity to mitochondrial dysfunction and oxidative stress (Wu et al., 2013). The latest research has reported that dopamine receptor neurons D1/D2 in the hippocampus affect dopamine release through different pathways, leading to emotion-related behaviors (Godino et al., 2025). Oxidative stress can cause mitochondrial dysfunction, leading to continuous accumulation of ROS, which further damages the synaptic plasticity and survival status of the dopaminergic neurons. Our experimental data showed that co-DR1/2I significantly upregulated MAO-B and ROS levels and reduced SOD activity, indicating that it may induce oxidative damage via mitochondrial dysfunction pathways. Previous studies have reported that MAO-B is involved in the neurotoxic response of MPTP mice (Sai et al., 2013). Oxidative stress has been proven to be the important pathogenic factor of dopaminergic neuron damage (Dionísio et al., 2021); however, oxidative stress can have a destructive effect on this originally relatively balanced state. When oxidative stress occurs, excessive ROS attacks DNA, RNA, proteins, etc., disrupting cellular homeostasis. Mitochondria, as the core source and main target of ROS, their functional damage further leads to ROS generation. Therefore, it interferes with the normal function of hippocampal dopaminergic neurons and has a negative impact on the nervous system. The results indicate that low-dose drugs have a greater oxidative stress effect, while high-dose drugs have a smaller oxidative stress effect. This may be because low-dose drugs activate adaptive antioxidant responses in cells, while high-dose drugs may directly overwhelm the defense system, leading to more severe effects, consistent with the poorer performance of the high-dose group in later behavioral experiments. This finding provides a new perspective and research direction for a deeper understanding about the regulatory mechanisms of the nervous system and e pathogenesis of related diseases. In depth research on this phenomenon would provide a theoretical basis for developing neuroprotective agents targeting the MAO-B/ROS pathway, which is expected to bring new breakthroughs and progress in the treatment and prevention of related diseases.

The effect of co-DR1/2I on dopaminergic neurons was reflected by the reduced number of TH-positive cells, directly indicating a decline in dopamine synthesis capacity. Notably, specific damage to dopaminergic neurons in the hippocampus was closely linked to spatial memory impairment in the water maze experiment, supporting the hypothesis that dopaminergic projections directly regulate cognitive function (Darvas and Palmiter, 2010). At the cellular level, early changes in oxidative stress indicators precede behavioral abnormalities, suggesting that MAO-B and ROS may serve as biomarkers for the early warning of neurotoxicity. Furthermore, differences in sensitivity to oxidative damage between the striatum and hippocampus, with the former exhibiting compensatory motor function and the latter showing cognitive deficits, reveal the functional heterogeneity of dopaminergic neuron subpopulations. These findings fill a gap in the understanding of the cooperative regulation of motor pathways by D1/D2 receptors while establishing their new role in cognitive-emotional circuits and providing experimental evidence at the cellular level for studying neurodegenerative disease mechanisms (Bonnavion et al., 2024).

Although this study did not directly measure immune indicators, the close association between oxidative stress and neuroinflammation suggests that co-DR1/2I may indirectly affect the neuroimmune environment. Increased MAO-B activity is known to promote the release of pro-inflammatory cytokines (such as IL-6 and TNF-α), while the accumulation of ROS can activate microglia, triggering a neuroinflammatory cascade (Kumar and Andersen, 2004; Bhoi et al., 2024, Jaisa-Aad et al., 2024; Venegas et al., 2024). The observed pattern of neuronal damage in the hippocampus is very similar to the “oxidative stress-inflammation-cell death” triad in neurodegenerative diseases (Liu et al., 2023), suggesting that co-DR1/2I may exacerbate neuroimmune imbalance through similar mechanisms. Notably, significant oxidative stress was observed even in the low-dose group, without obvious inflammatory infiltration, which may reflect an early barrier effect of the blood-brain barrier on immune cell migration. These findings provide new insights into the crosstalk between dopamine receptor regulation and neuroimmunity and offer important implications for developing combined therapies with both antipsychotic and neuroprotective effects.

This study has some limitations that need to be noted. First, the sample size in each group may have affected the statistical power, particularly limiting the interpretability of behavioral tests with significant individual differences (such as the open-field experiment). Second, the study focused solely on the dopaminergic system and did not assess compensatory changes in other neurotransmitter networks, such as 5-HT and GABA, which may lead to a one-sided understanding of the mechanisms. Third, the 28-days dosing period, while allowing the observation of acute effects, did not assess the potential long-term neuroadaptive changes or tardive motor symptoms that may arise from prolonged medication. Additionally, the study used traditional techniques, such as ELISA and immunofluorescence, but it lacked molecular pathway analysis at the transcriptomic or other proteomic proteomic level, making it difficult to reveal the regulatory network upstream of oxidative stress. In the future, we will consider extending the duration of drug action, comparing changes in oxidative stress markers after prolonged use and detecting downstream signaling pathway proteins to clarify the specific mechanism of action of co-DR1/2I on dopamine neurons and its impact on related neurotransmitters.

In summary, this study confirms that co-DR1/2I induces dopaminergic neuronal damage through an imbalance in the MAO-B-ROS-SOD axis, leading to cognitive and emotional dysfunction. Notably, the most significant changes in oxidative stress indicators were observed in the low-dose group, whereas behavioral abnormalities were relatively mild, suggesting the existence of a neuro-compensatory window, which provides a theoretical basis for developing early intervention strategies for neurological diseases. Future research should focus on exploring combined treatment regimens targeting antioxidants, receptor modulators, and toxicity warning systems based on region-specific biomarkers.

## Data Availability

The original contributions presented in this study are included in this article/Supplementary material, further inquiries can be directed to the corresponding author.
