# Supplementary figures and images for: Combined inhibition of dopamine D1/D2 receptors induces cognitive and emotional dysfunction through oxidative stress and dopaminergic neuron damage
Source: Front Behav Neurosci. 2025 Aug 4;19:1621017. doi: 10.3389/fnbeh.2025.1621017 (PMC12358472; doi:10.3389/fnbeh.2025.1621017)

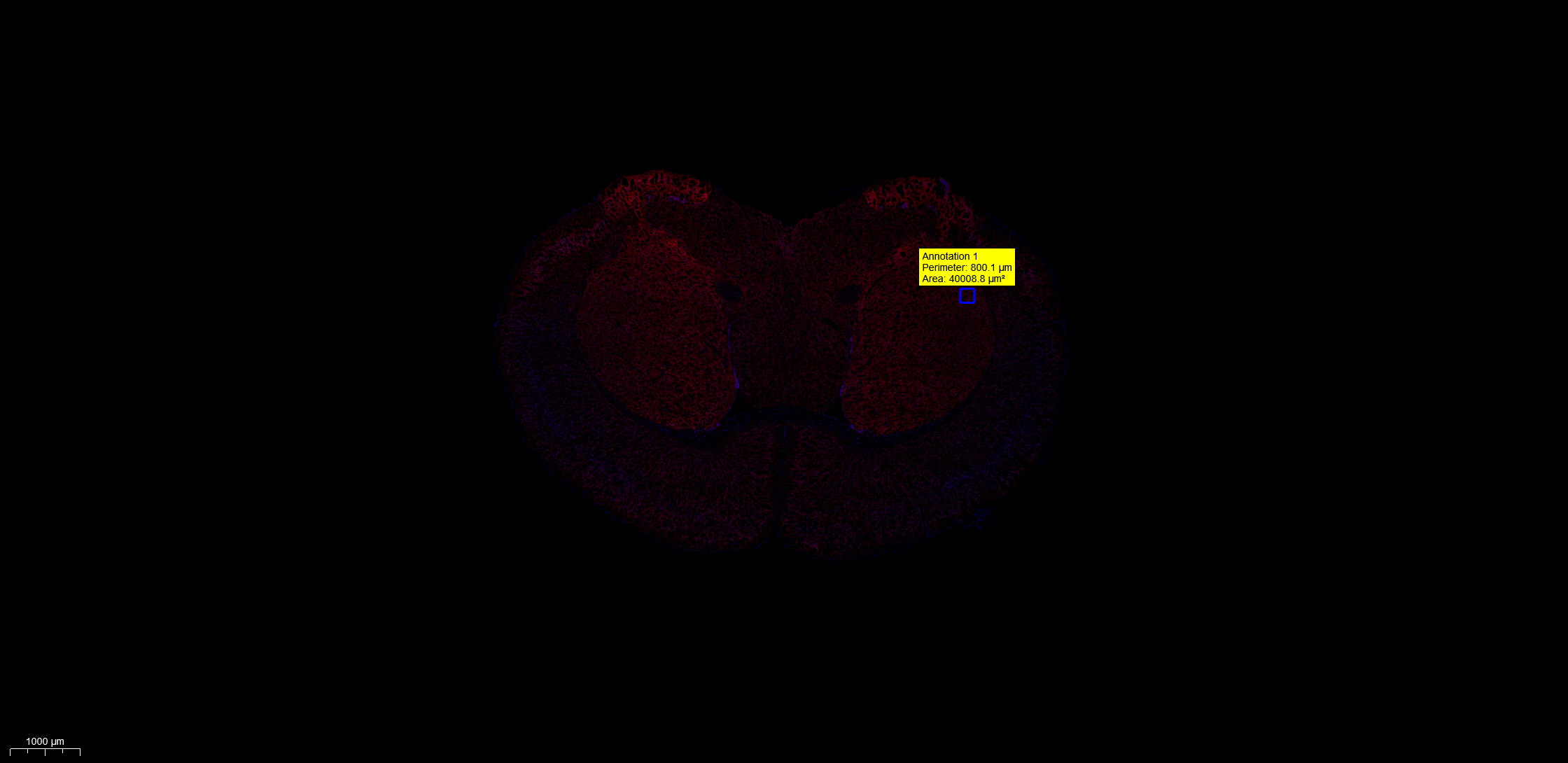

Supplement: Supplementary file 1 [file Data_Sheet_1.zip › control/control- CPU- TH_2.0x-whole_scan.jpg]

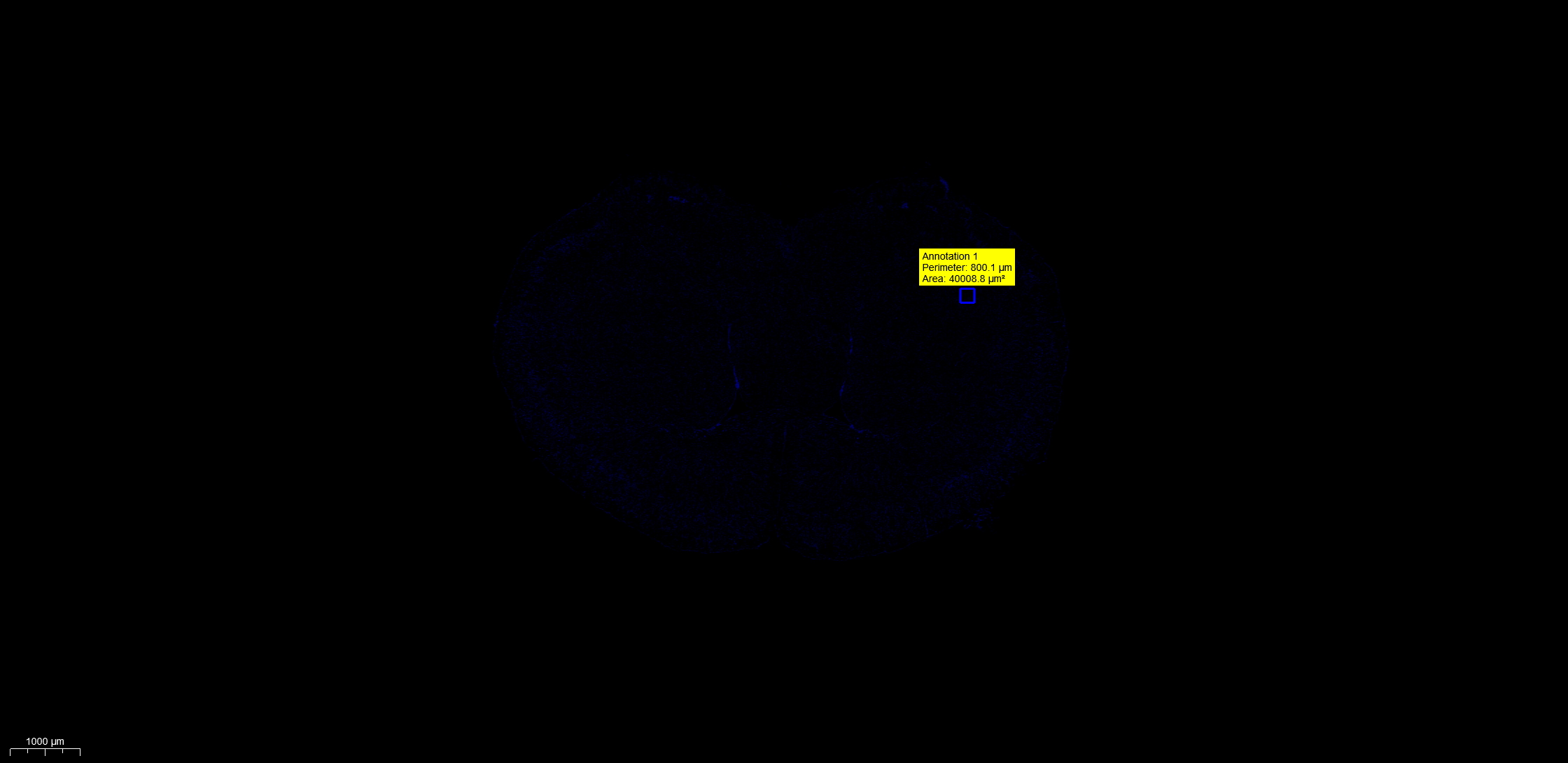

Supplement: Supplementary file 1 [file Data_Sheet_1.zip › control/control- CPU- TH_2.0xDAPI.jpg]

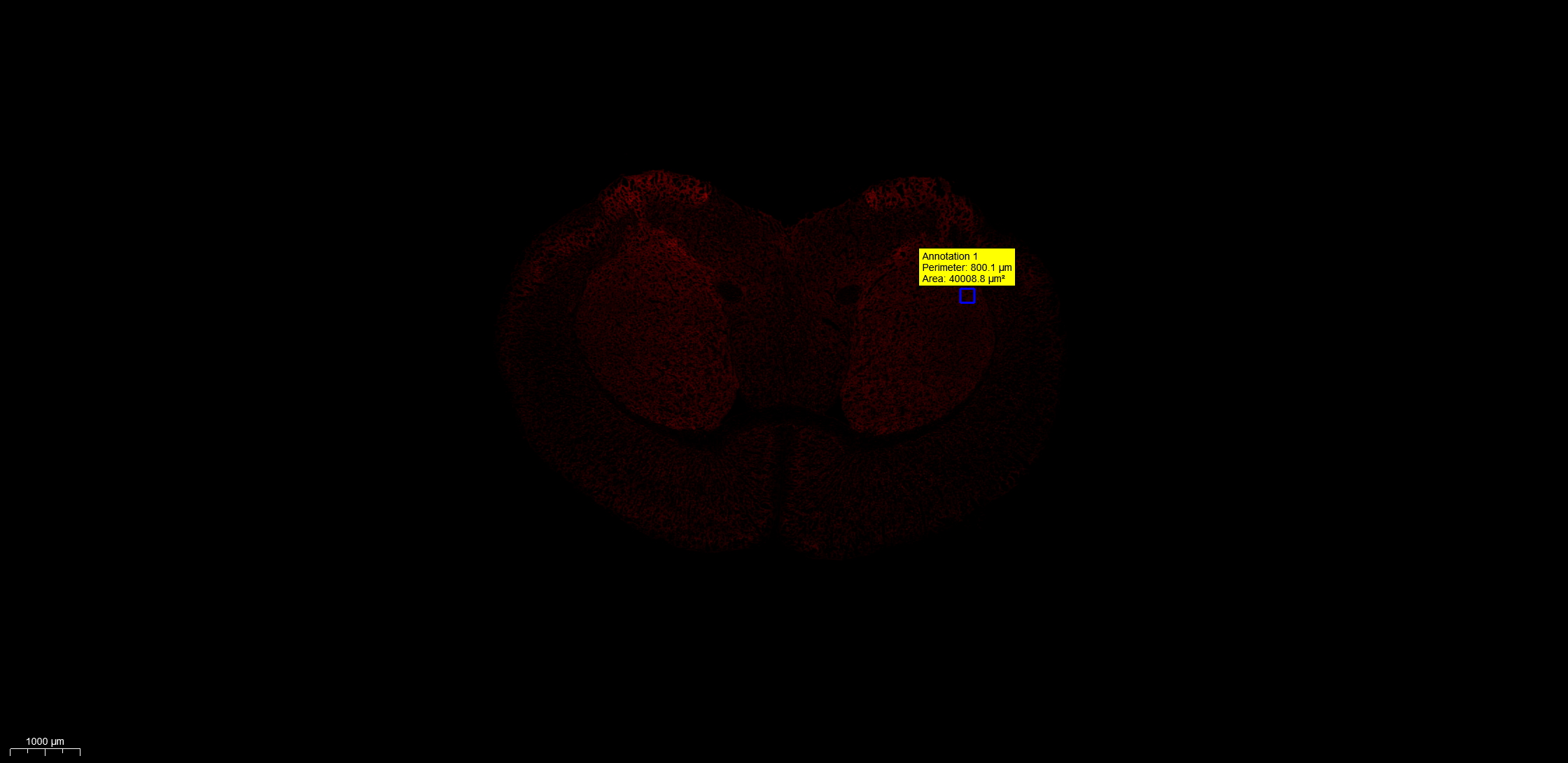

Supplement: Supplementary file 1 [file Data_Sheet_1.zip › control/control- CPU- TH_2.0xSporange.jpg]

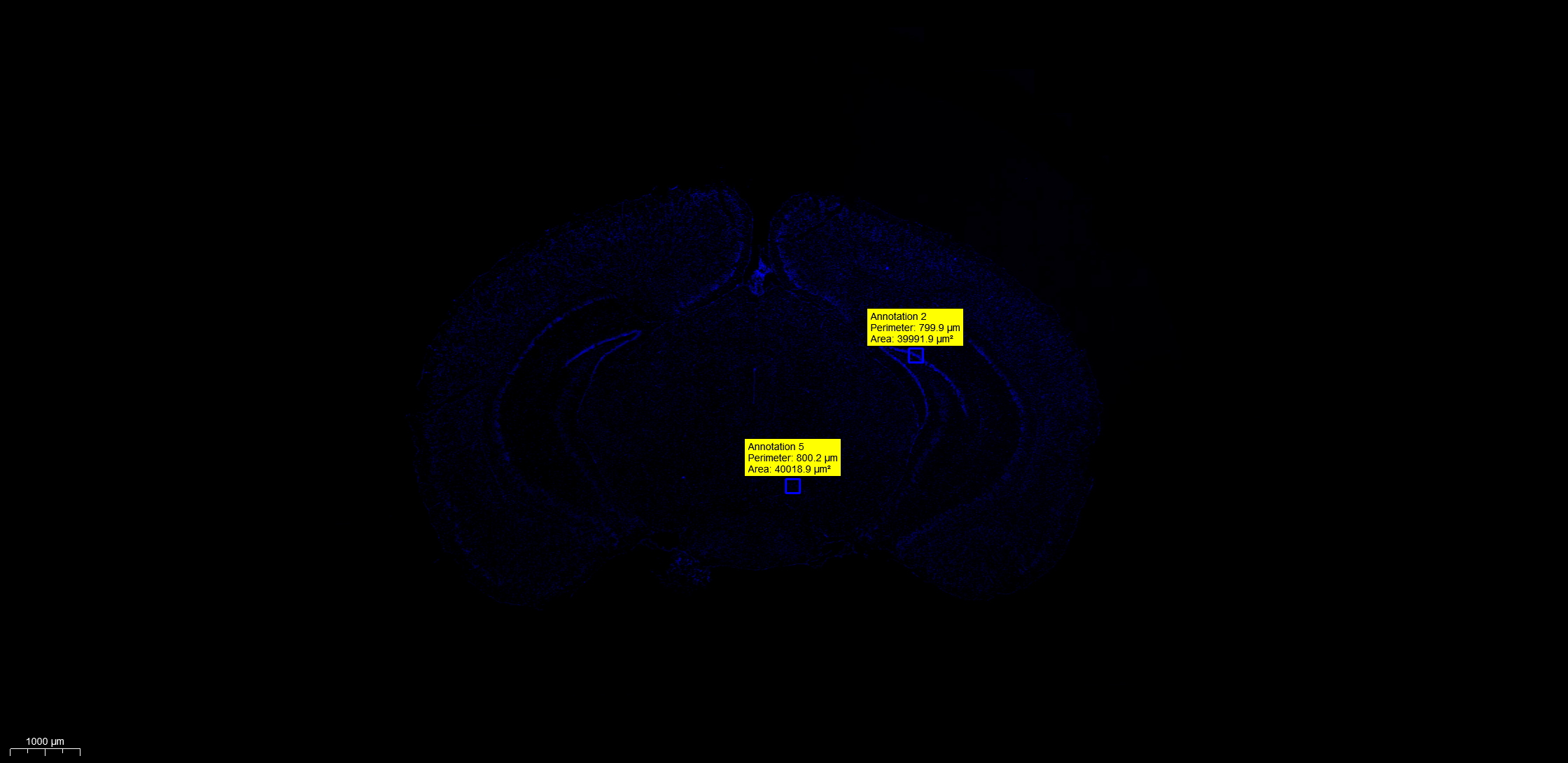

Supplement: Supplementary file 1 [file Data_Sheet_1.zip › control/control- SN.Hi- TH_2.0x-DAPI.jpg]

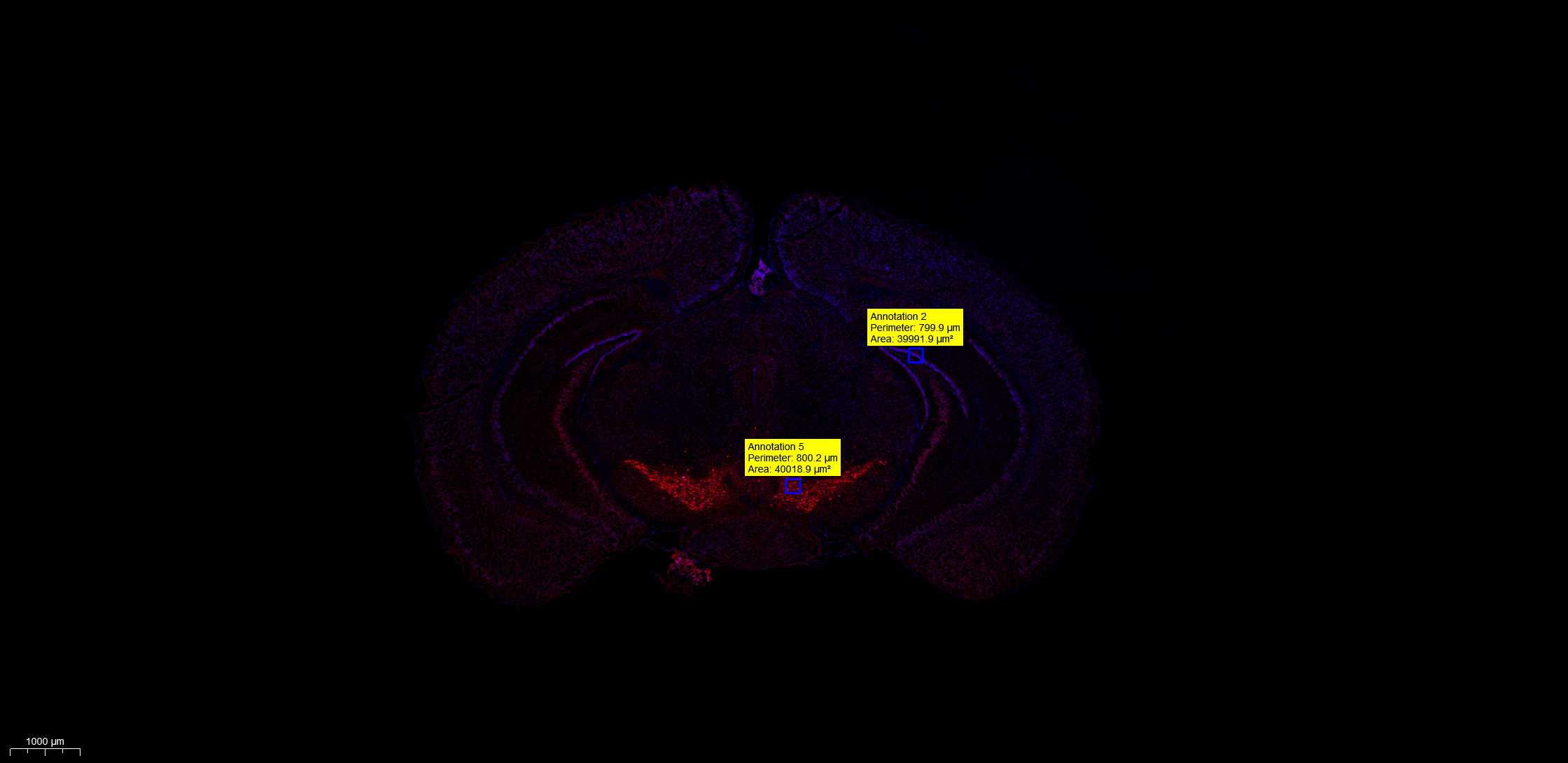

Supplement: Supplementary file 1 [file Data_Sheet_1.zip › control/control- SN.Hi- TH_2.0x-whole_scan.jpg]

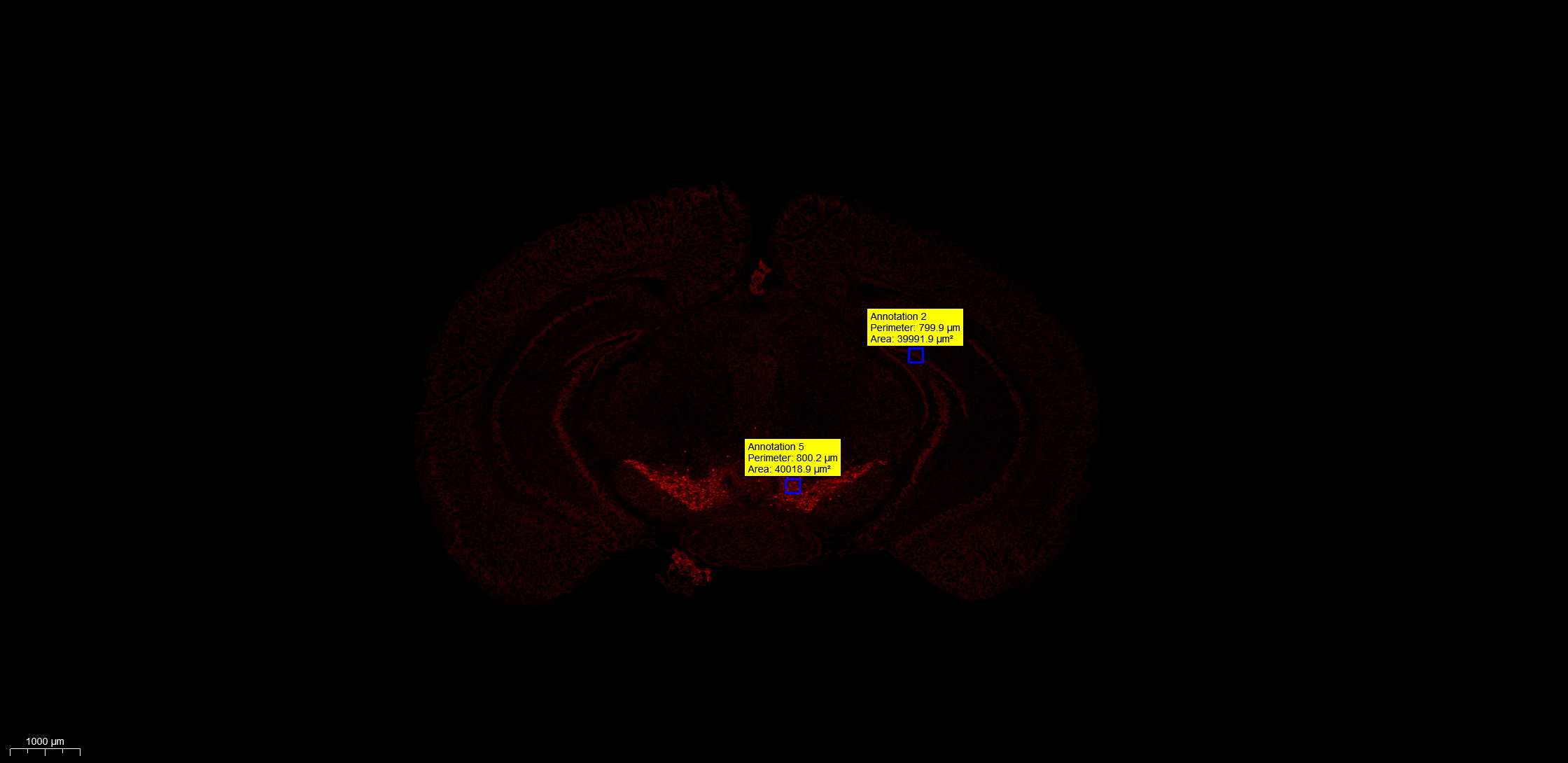

Supplement: Supplementary file 1 [file Data_Sheet_1.zip › control/control- SN.Hi- TH_2.0xSporange.jpg]

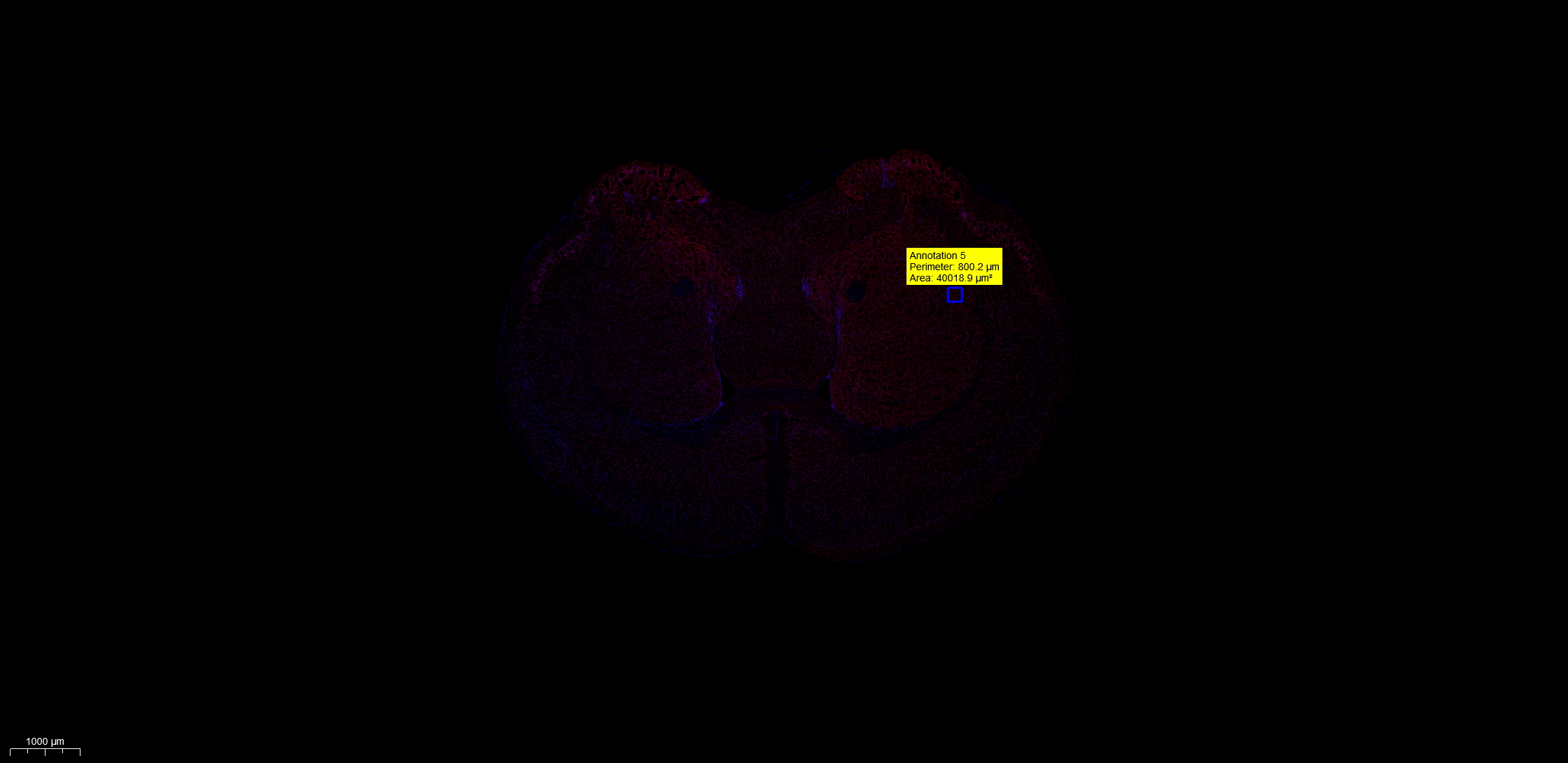

Supplement: Supplementary file 2 [file Data_Sheet_2.zip › Hco-DR12I/Hco-DR12I-CPU- TH_2.0x-whole_scan.jpg]

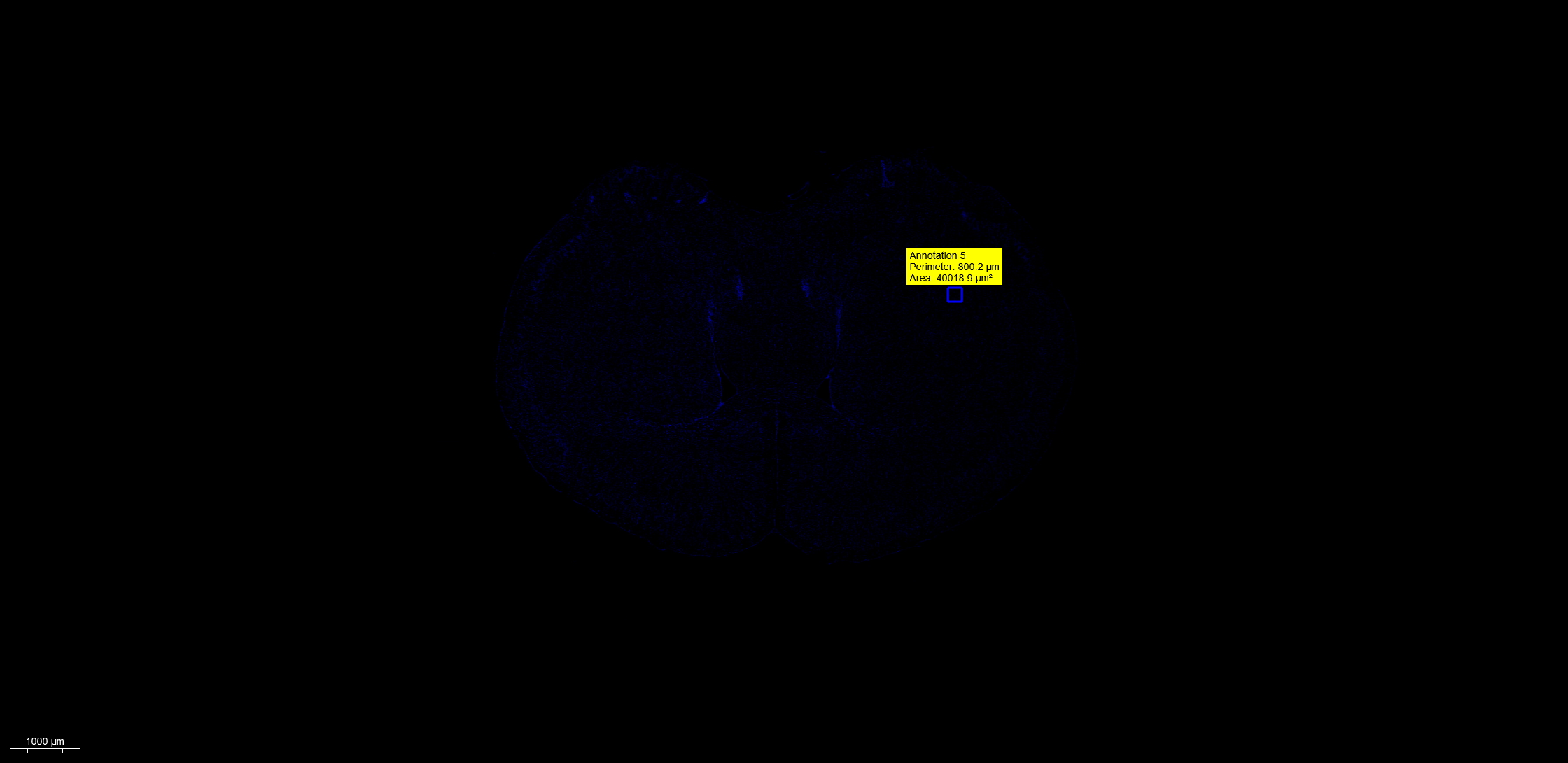

Supplement: Supplementary file 2 [file Data_Sheet_2.zip › Hco-DR12I/Hco-DR12I-CPU- TH_2.0xDAPI.jpg]

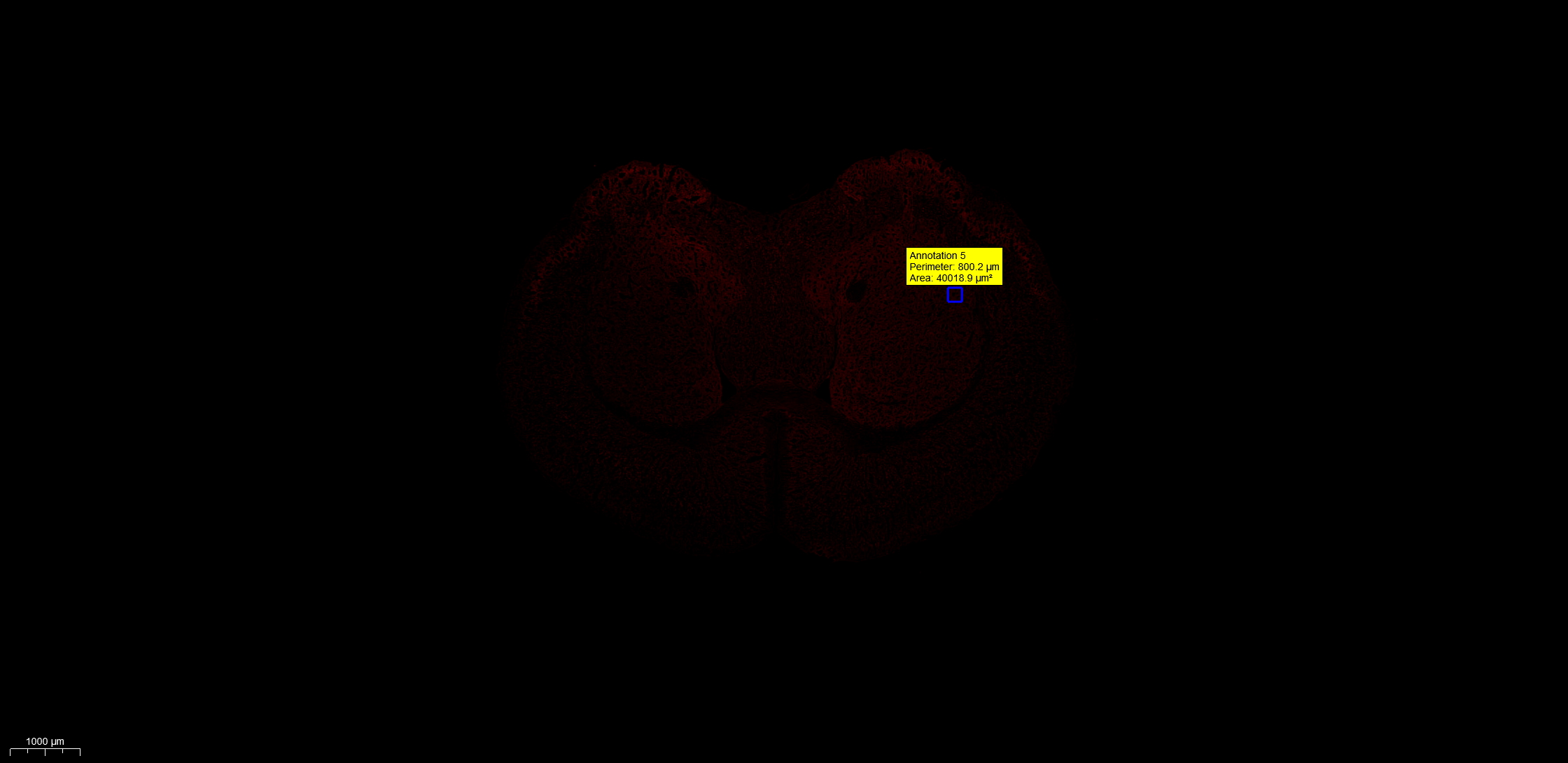

Supplement: Supplementary file 2 [file Data_Sheet_2.zip › Hco-DR12I/Hco-DR12I-CPU- TH_2.0xSporange.jpg]

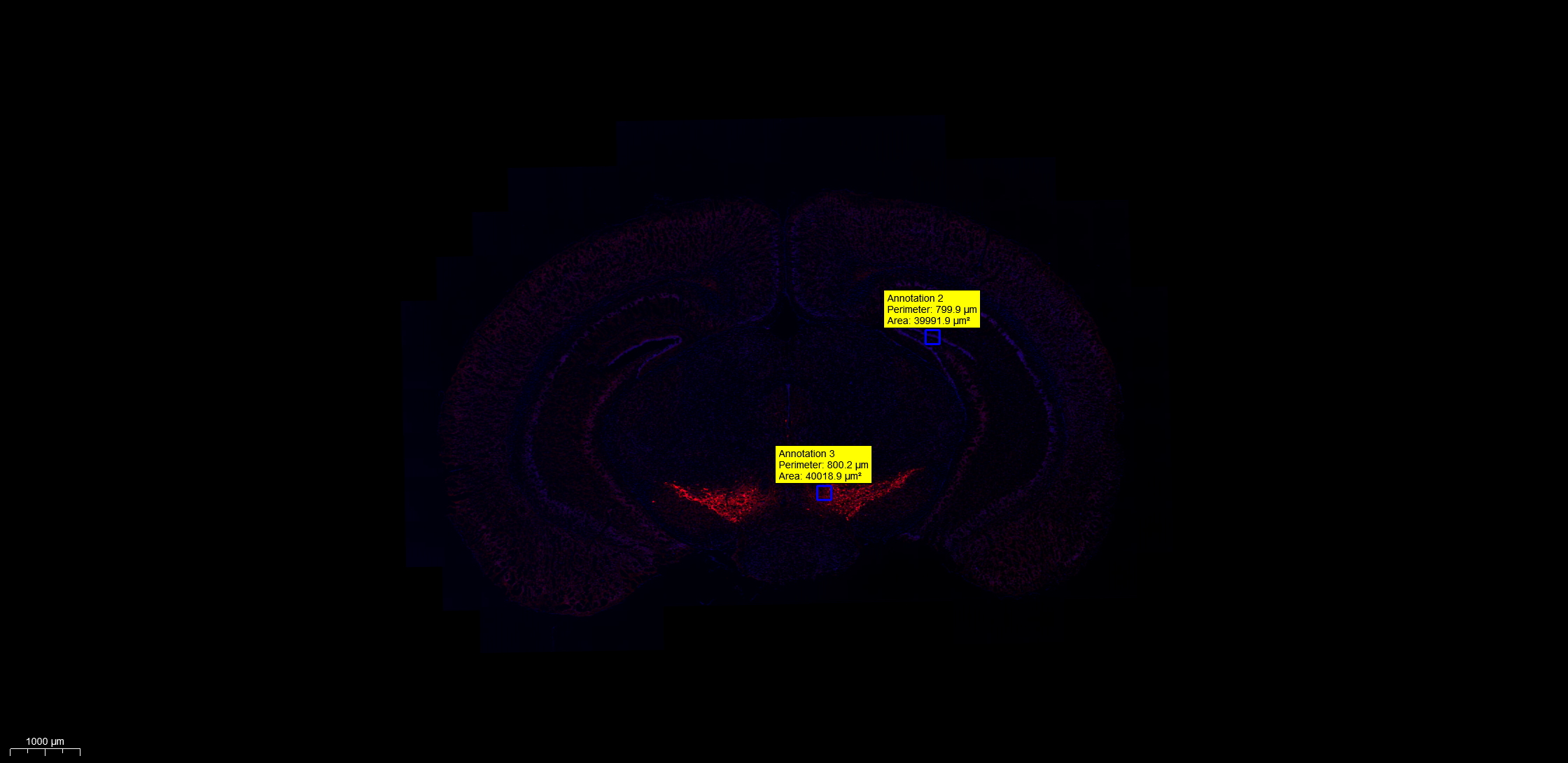

Supplement: Supplementary file 2 [file Data_Sheet_2.zip › Hco-DR12I/Hco-DR12I-SN.Hi- TH_2.0x-whole_scan.jpg]

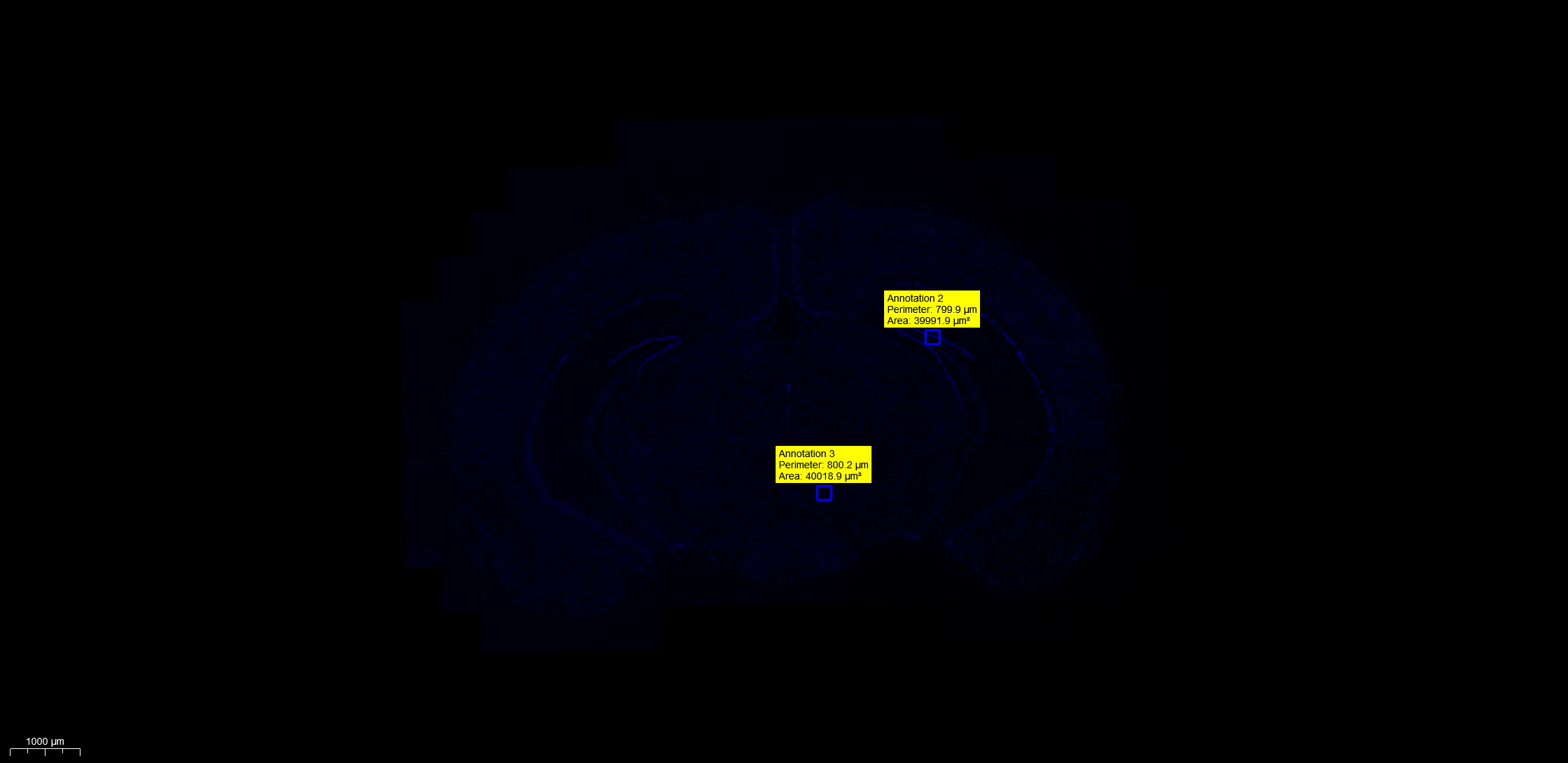

Supplement: Supplementary file 2 [file Data_Sheet_2.zip › Hco-DR12I/Hco-DR12I-SN.Hi- TH_2.0xDAPI.jpg]

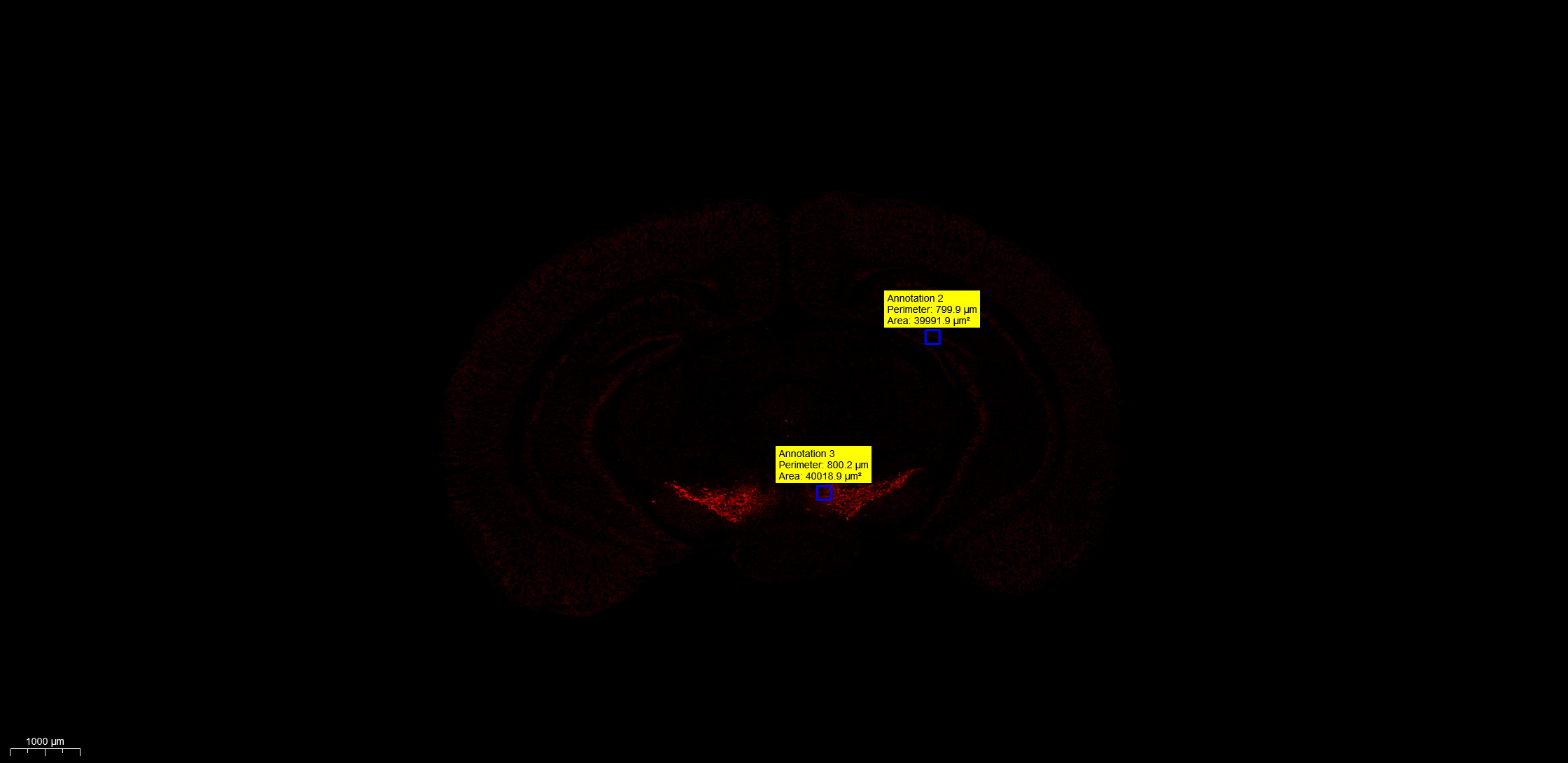

Supplement: Supplementary file 2 [file Data_Sheet_2.zip › Hco-DR12I/Hco-DR12I-SN.Hi- TH_2.0xSporange.jpg]

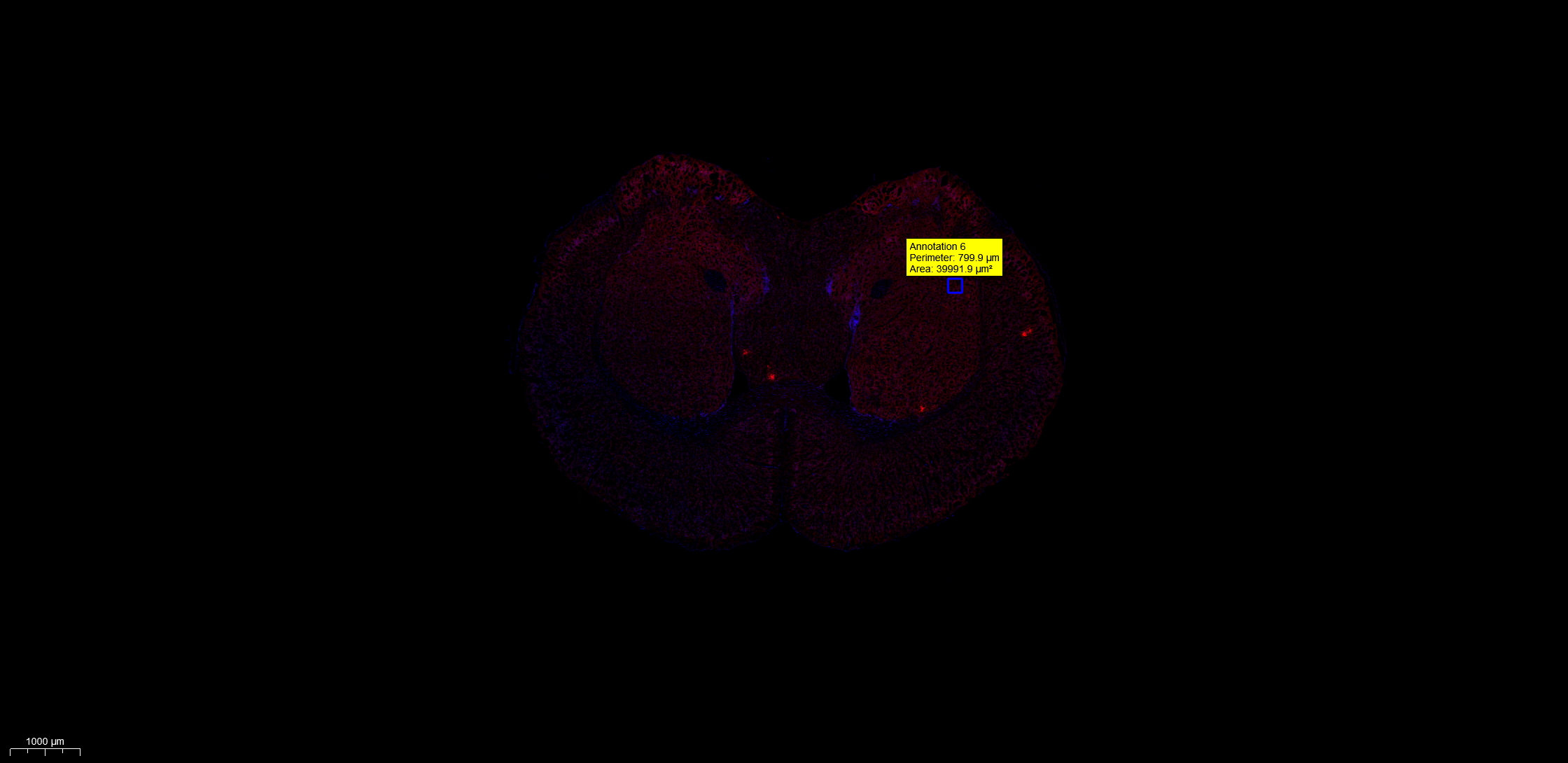

Supplement: Supplementary file 3 [file Data_Sheet_3.zip › Lco-DR12I/Lco-DR12I- CPU-TH_2.0x-whole_scan.jpg]

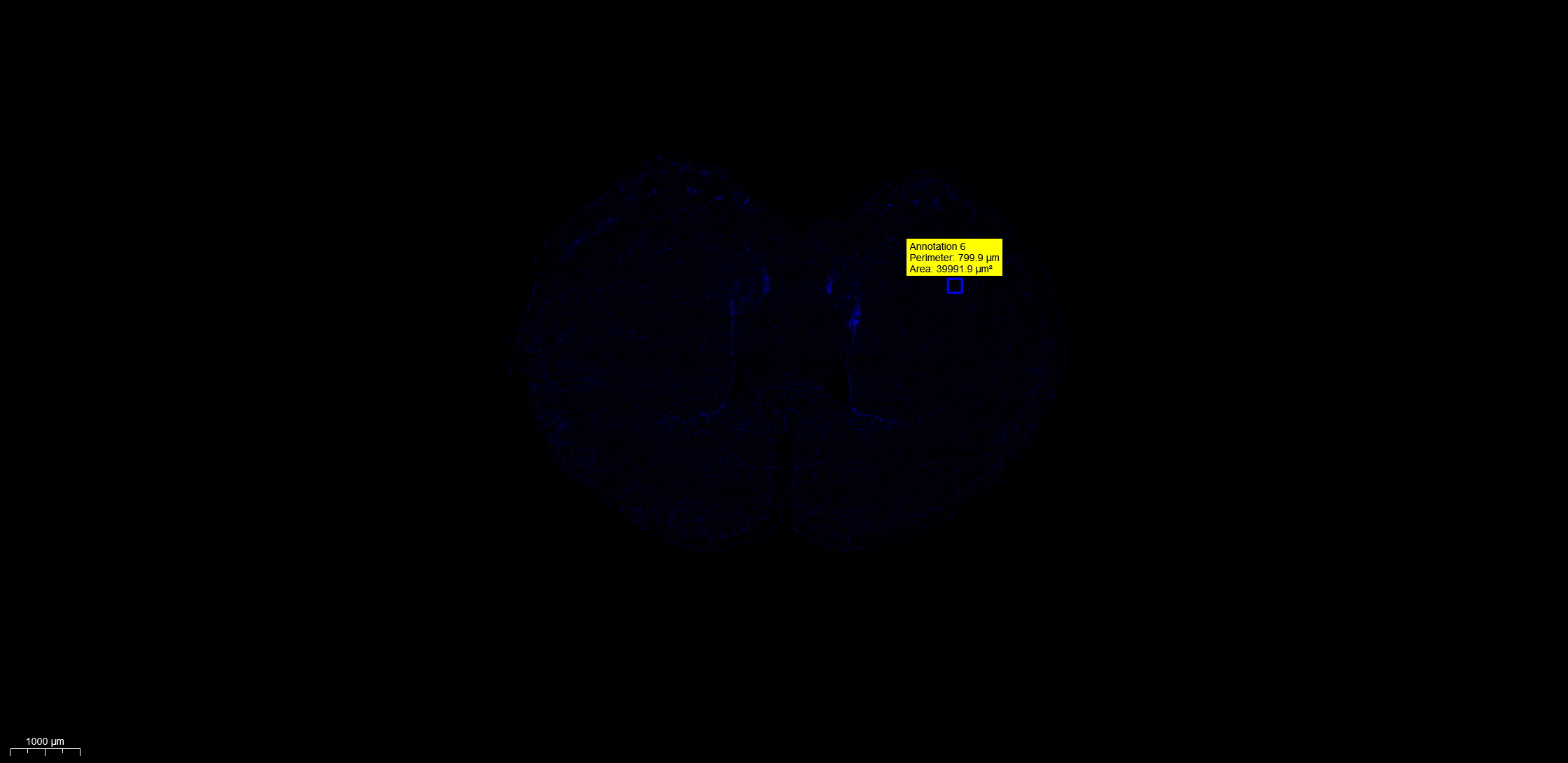

Supplement: Supplementary file 3 [file Data_Sheet_3.zip › Lco-DR12I/Lco-DR12I- CPU-TH_2.0xDAPI.jpg]

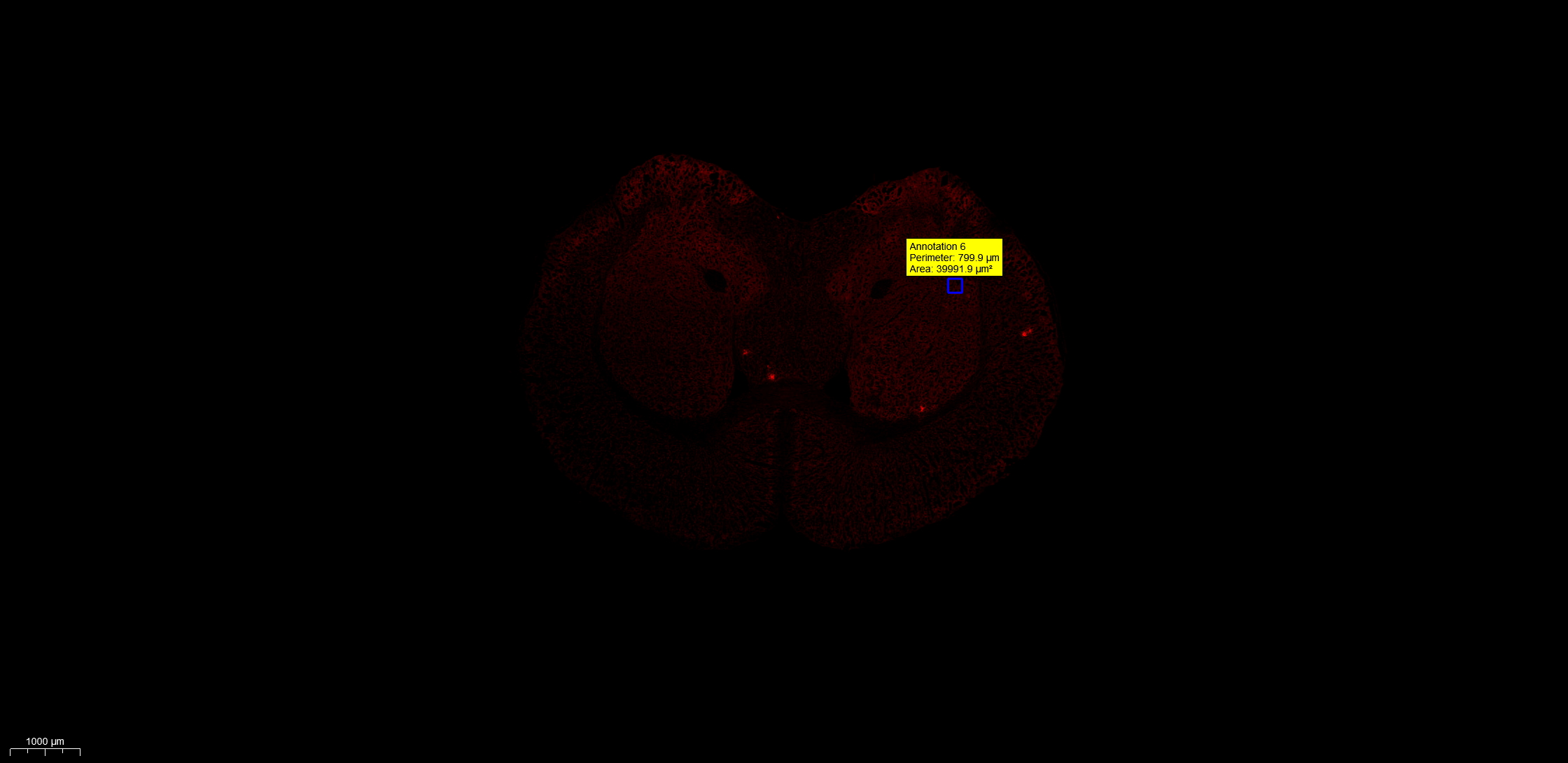

Supplement: Supplementary file 3 [file Data_Sheet_3.zip › Lco-DR12I/Lco-DR12I- CPU-TH_2.0xSporange.jpg]

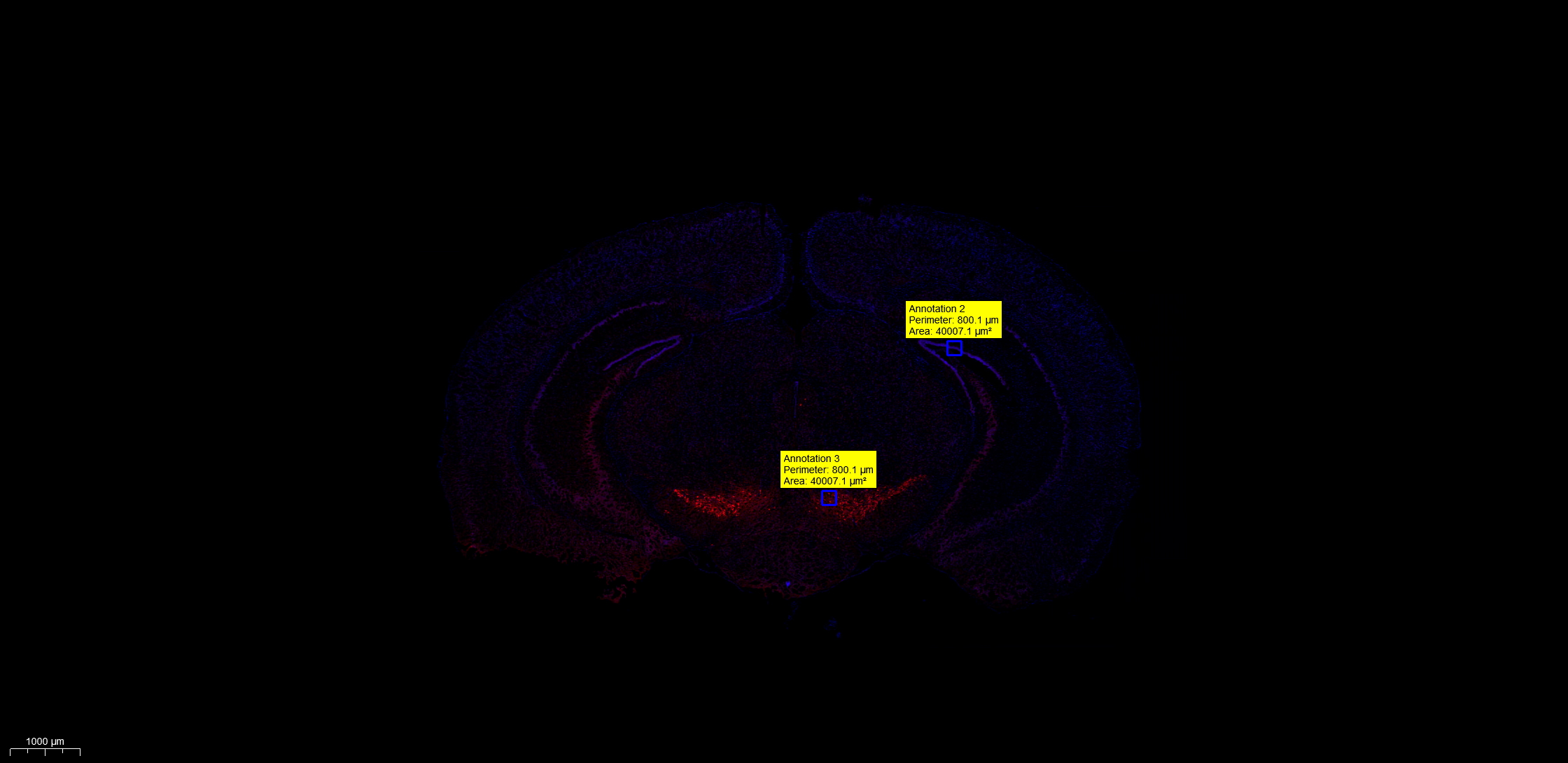

Supplement: Supplementary file 3 [file Data_Sheet_3.zip › Lco-DR12I/Lco-DR12I-SN.Hi- TH_2.0x-whole_scan.jpg]

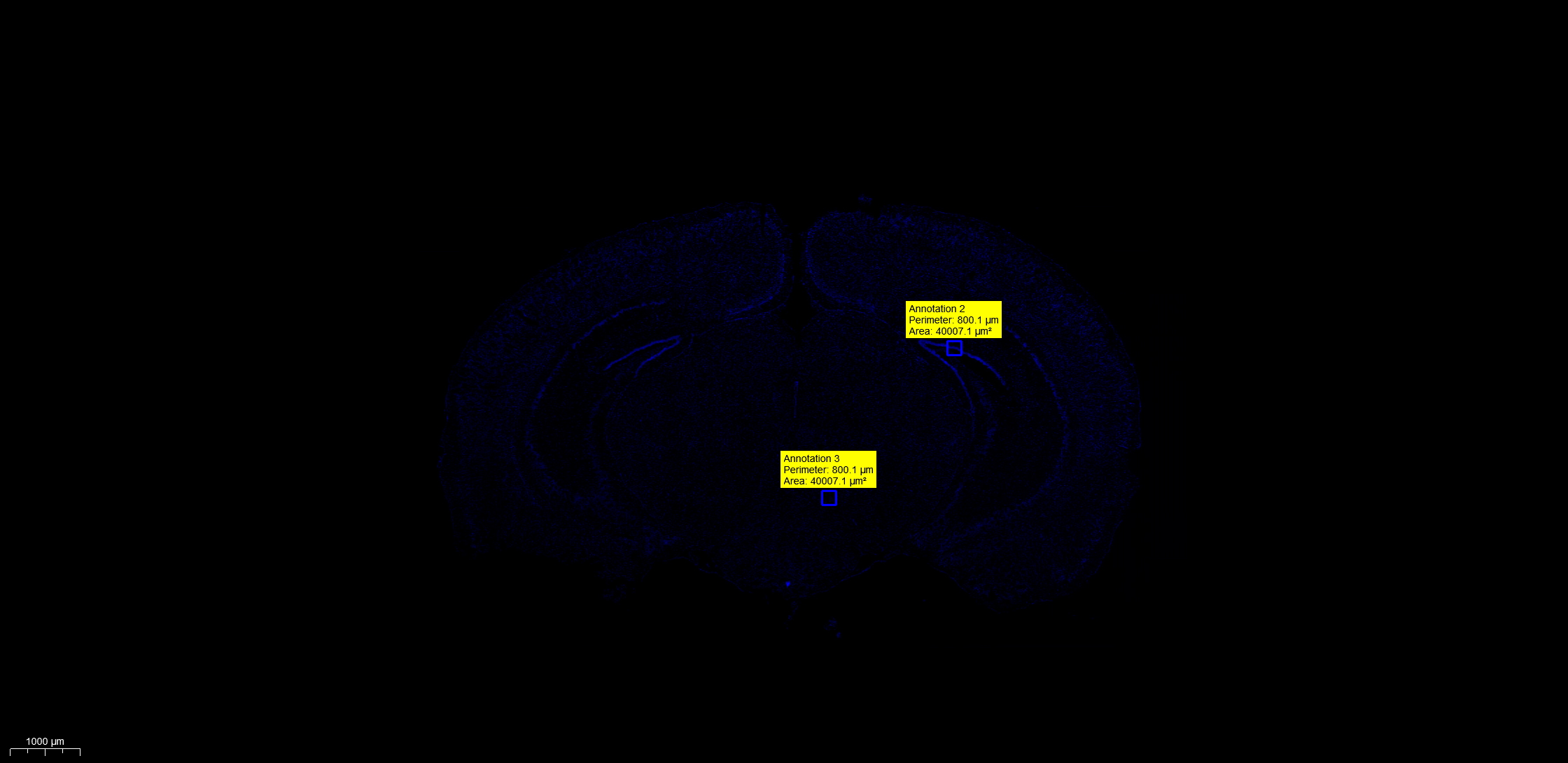

Supplement: Supplementary file 3 [file Data_Sheet_3.zip › Lco-DR12I/Lco-DR12I-SN.Hi- TH_2.0xDAPI.jpg]

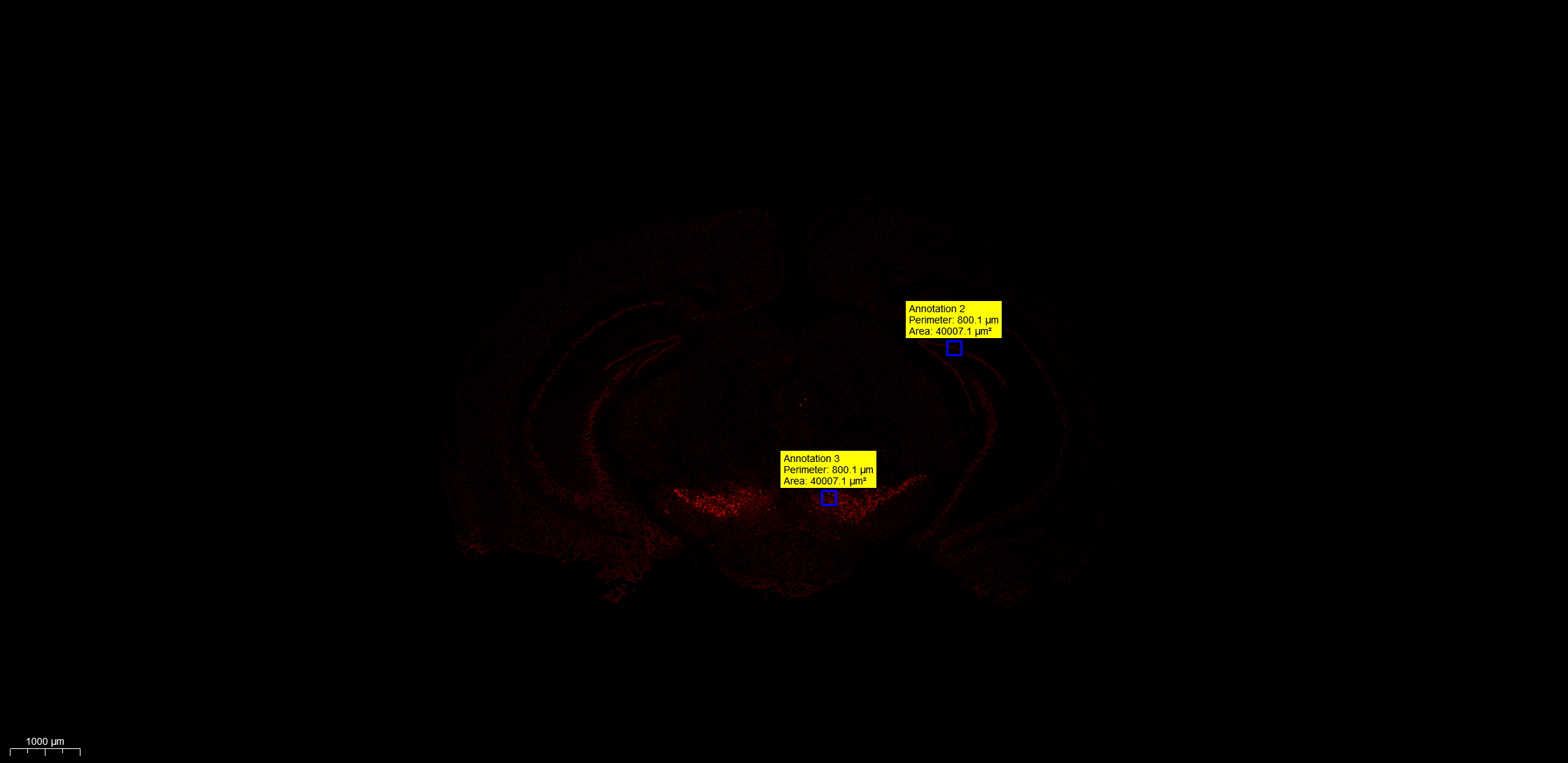

Supplement: Supplementary file 3 [file Data_Sheet_3.zip › Lco-DR12I/Lco-DR12I-SN.Hi- TH_2.0xSporange.jpg]

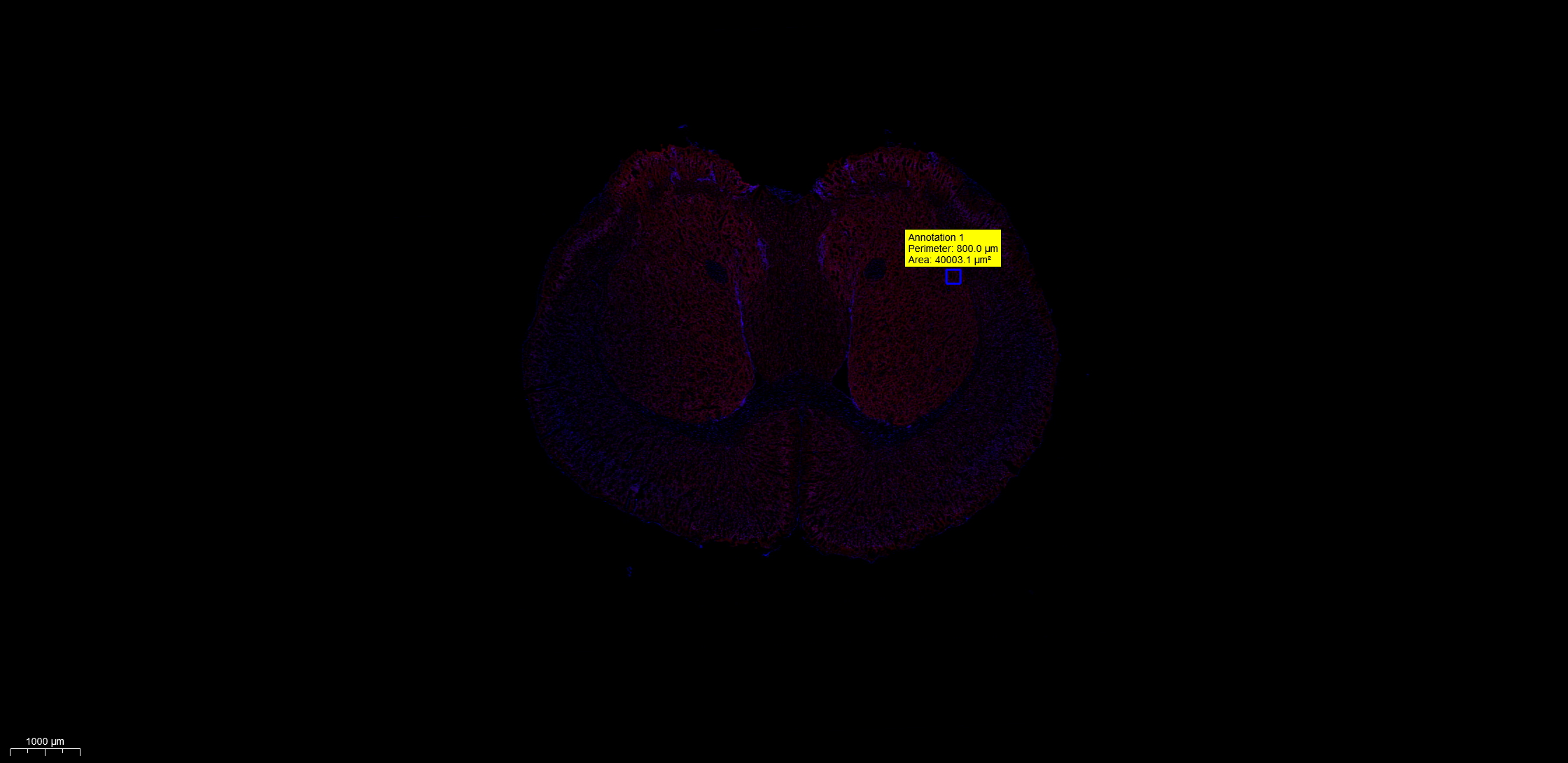

Supplement: Supplementary file 4 [file Data_Sheet_4.zip › Mco-DR12I/Mco-DR12I-CPU- TH_2.0x-whole_scan.jpg]

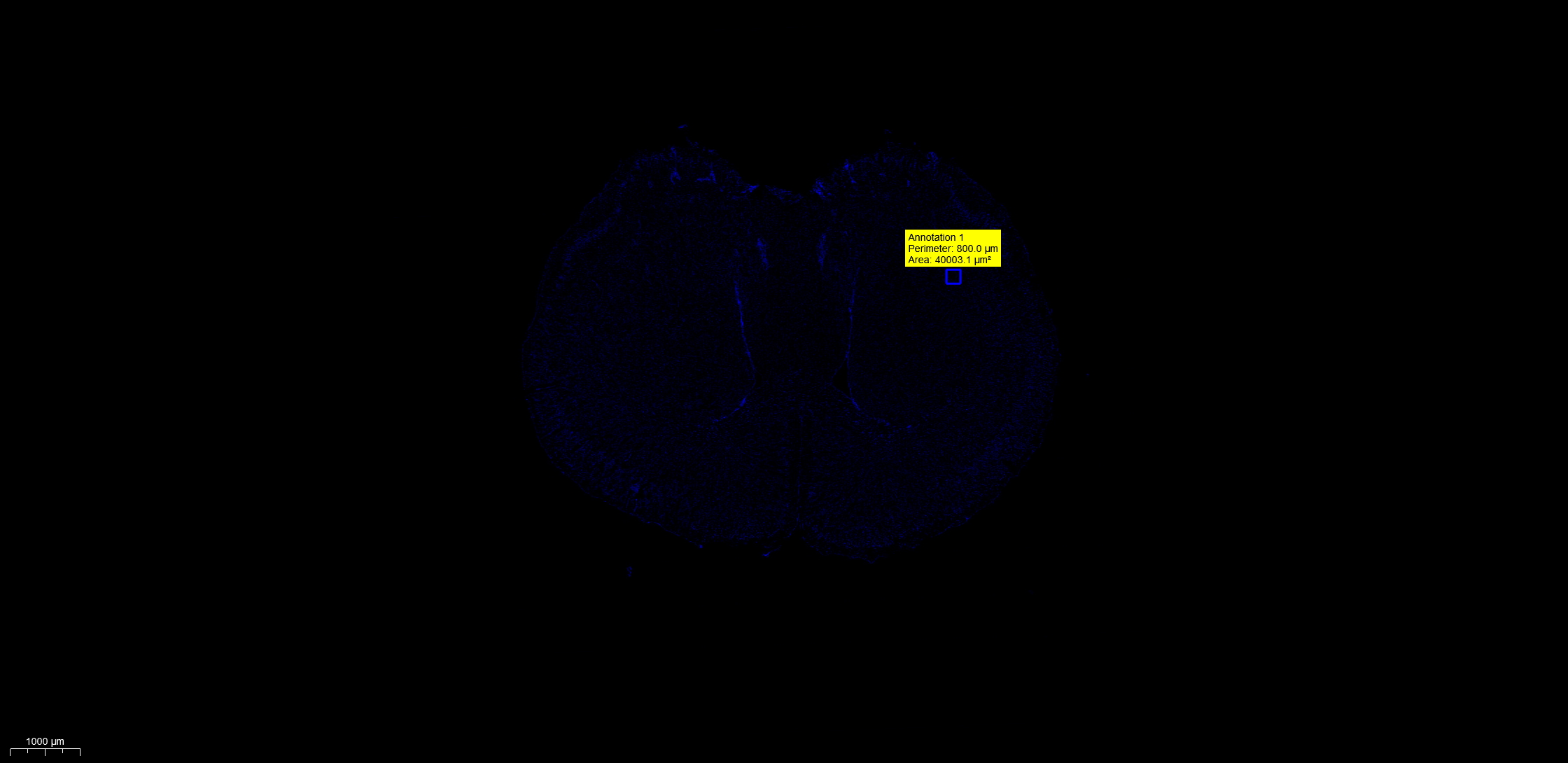

Supplement: Supplementary file 4 [file Data_Sheet_4.zip › Mco-DR12I/Mco-DR12I-CPU- TH_2.0xDAPI.jpg]

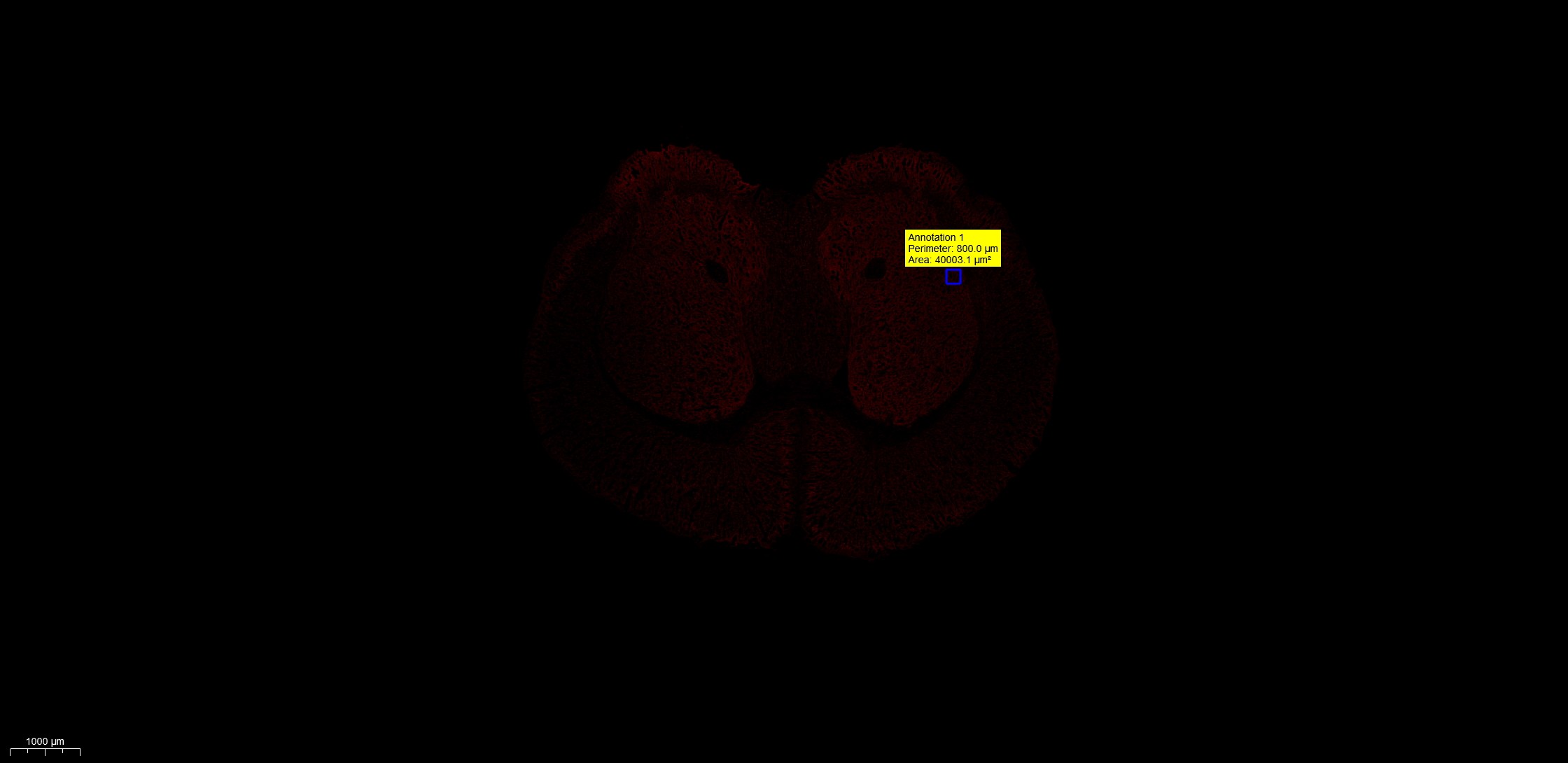

Supplement: Supplementary file 4 [file Data_Sheet_4.zip › Mco-DR12I/Mco-DR12I-CPU- TH_2.0xSporange.jpg]

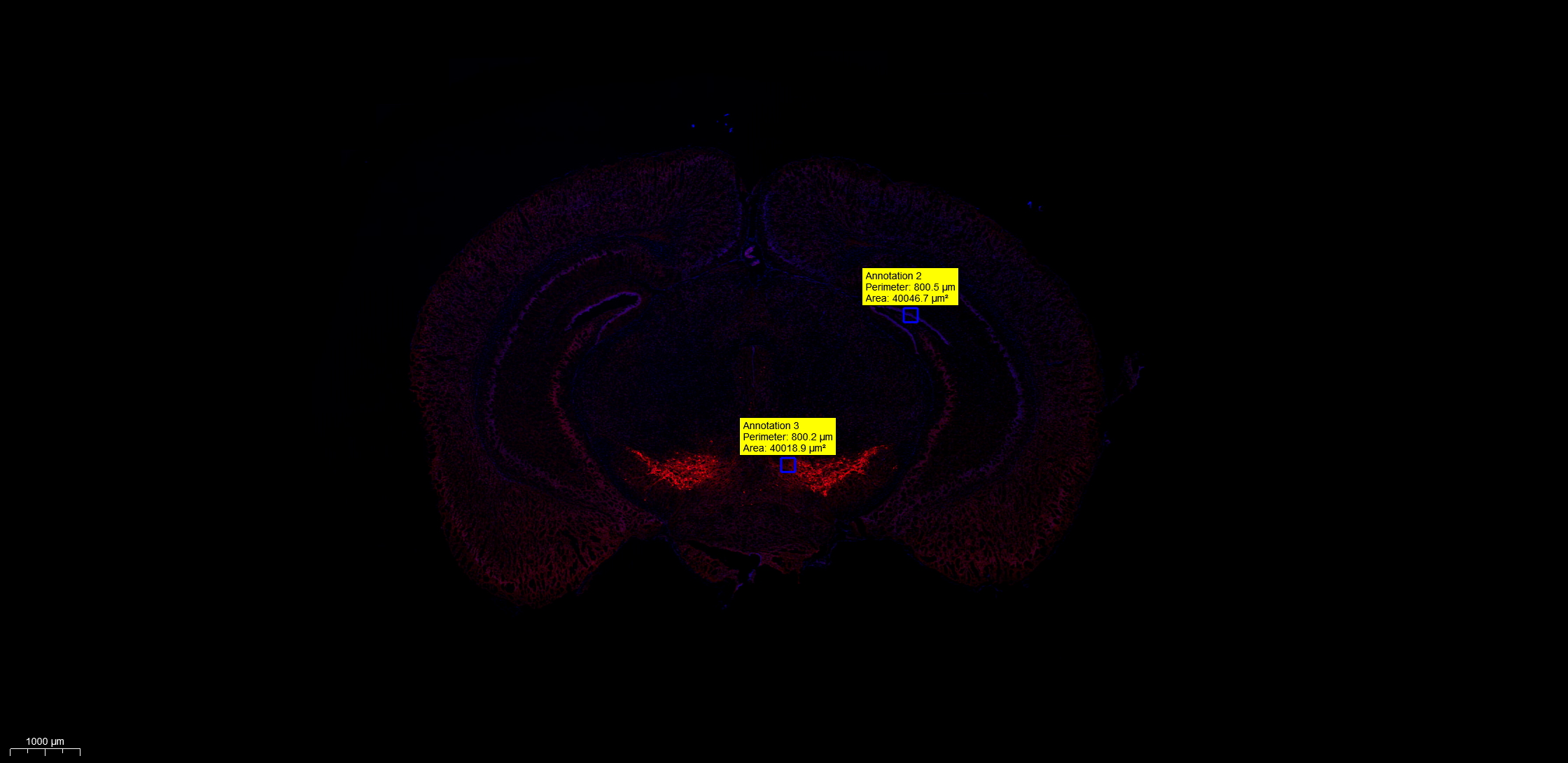

Supplement: Supplementary file 4 [file Data_Sheet_4.zip › Mco-DR12I/Mco-DR12I-SN.Hi- TH_2.0x-whole_scan.jpg]

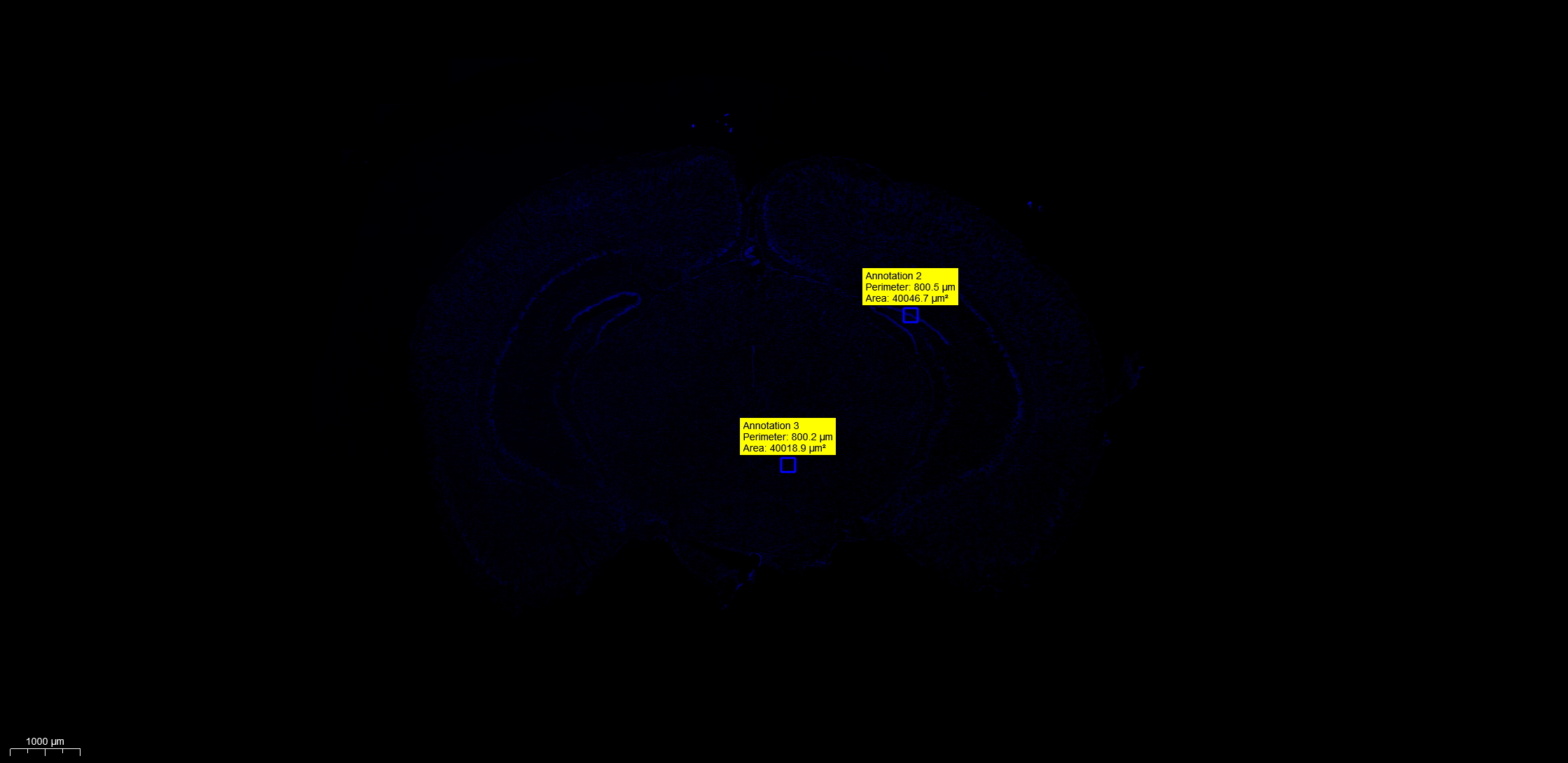

Supplement: Supplementary file 4 [file Data_Sheet_4.zip › Mco-DR12I/Mco-DR12I-SN.Hi- TH_2.0xDAPI.jpg]

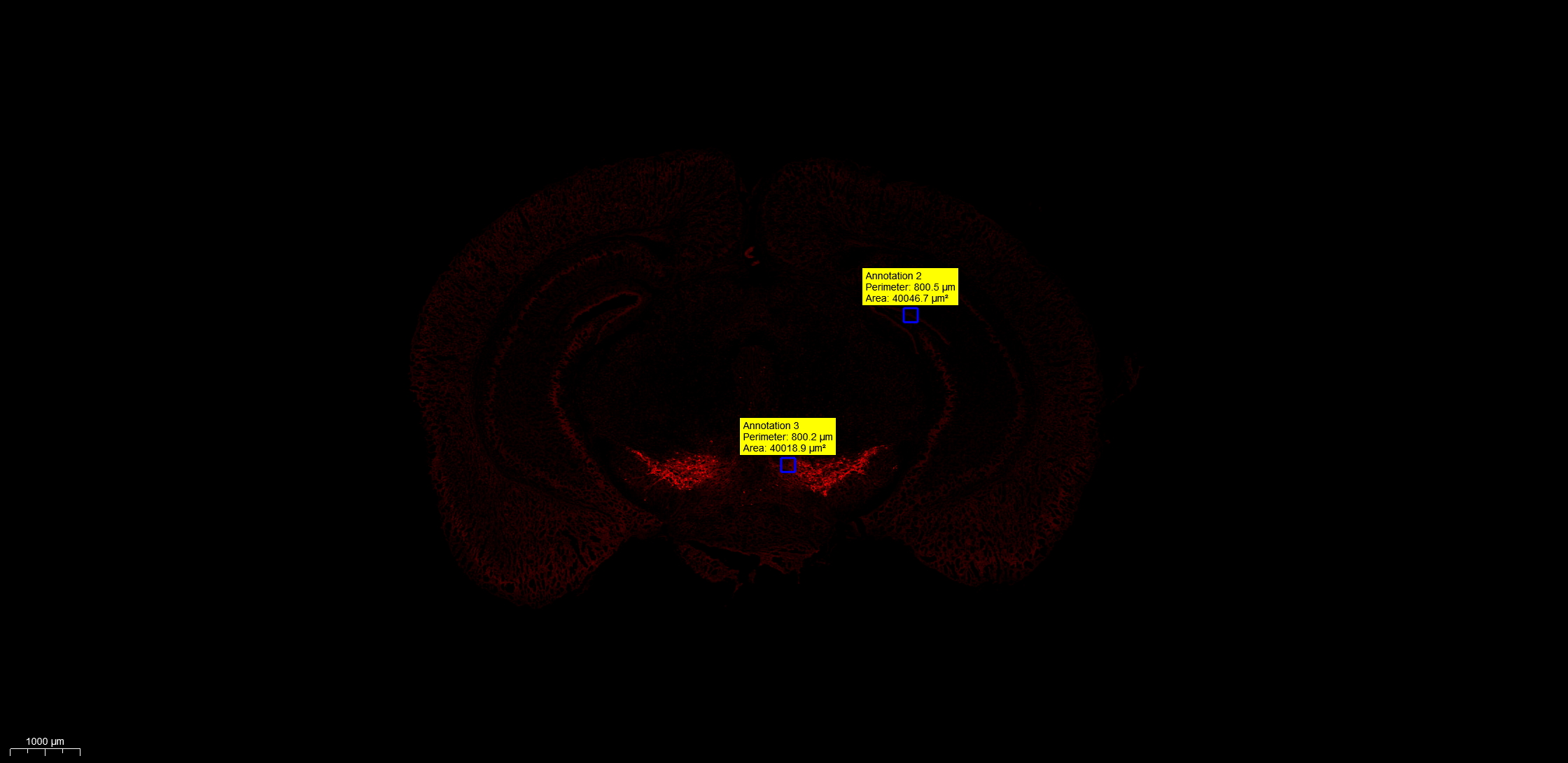

Supplement: Supplementary file 4 [file Data_Sheet_4.zip › Mco-DR12I/Mco-DR12I-SN.Hi- TH_2.0xSporange.jpg]
